# Impact of Mobilization Facilitated by Wearable Device Enhanced Patient Monitoring/Electrophysiology Pod–Based Feedback on Postoperative Complications Following Colorectal Cancer Surgery: Randomized Controlled Trial

**DOI:** 10.2196/70534

**Published:** 2026-01-30

**Authors:** Yang Meng, Fengyan Fan, Yumei Ma, Nong Yan, Huan Wang, Zhen Zhang, Yiting Wang, Hailong Dong, Huang Nie

**Affiliations:** 1 Department of Anesthesiology and Perioperative Medicine, Fourth Military Medical University Xijing Hospital, No.127 Changle West Road, Xi'an, 710032, China, 86 13201630298; 2 Department of Anesthesiology, No.905 Hospital of PLA NAVY, Shanghai, China; 3 Mindray Medical International Ltd, Shenzhen, China

**Keywords:** wearable device, postoperative complications, ERAS, colorectal cancer, postoperative mobilization, enhanced recovery after surgery

## Abstract

**Background:**

Enhanced recovery after surgery (ERAS) guidelines recommend early postoperative mobilization to reduce complications, but adherence is often suboptimal, highlighting the need for effective tools to monitor and encourage movement. The Mindray enhanced patient monitoring (ePM)/electrophysiology (ep) pod, capable of tracking activity, vital signs, sleep, and pain, offers high-precision postoperative monitoring and is well-suited for research on activity feedback.

**Objective:**

The study aims to assess whether wearable device-based (ePM/ep pod) activity feedback could reduce postoperative complications within 30 days of colorectal cancer (CRC) surgery.

**Methods:**

We conducted an open-label, evaluator-blind, randomized controlled trial involving patients aged ≥18 years scheduled for CRC surgery. Patients were randomized to a feedback group or a control group. Both groups were set the same target activity time postoperatively based on ERAS guidelines. The feedback group received real-time visual feedback of movement time daily through the ePM/ep pod device, while the control group did not receive feedback. The primary outcome was the comprehensive complication index (CCI) within postoperative 30 days. Secondary outcomes included daily activity time, pain Numeric Rating Scale scores for rest and movement during the first 3 postoperative days, length of stay, percentage of reaching the scheduled mobilization target, 30-day postoperative mortality rate, and the times of first exhaust and defecation.

**Results:**

Two hundred thirty-nine patients were recruited between February 2023 and September 2023, with 216 randomized (n=108 for each group). There was no significant difference in CCI within 30 postoperative days between the control group (median CCI 0, range 0‐20.90) and the activity feedback group (median CCI 0, range 0‐12.20). The estimated mean difference was −0.59 (95% CI −3.56 to 2.38; *P*=.66). Sensitivity analysis excluding patients with low device compliance did not alter these findings. No significant differences between groups were found in daily activity time, length of hospital stay, or pain scores. Post hoc analysis revealed significant negative correlations between 30-day CCI and activity on the second day after operation (*r*=−0.166) and the third day after operation (POD3) (*r*=−0.264; *P*<.05 for both). Linear regression indicated that POD3 activity significantly reduced CCI (*β*=−.025; 95% CI −0.045 to −0.006*; P*=.01), with peak CCI reduction at 215 minutes of activity.

**Conclusions:**

In the context of ERAS, this study found no evidence that activity stimulation based on feedback from the wearable device (ePM/ep pod) could reduce 30-day postoperative CCI in patients undergoing CRC surgery. However, the ePM/ep pod could accurately record daily activity duration, which may be negatively correlated with CCI on POD3.

## Introduction

Colorectal cancer (CRC) is the second most common cancer in China and ranks third globally in mortality rate [1,2], which is an urgent health problem. Surgery is the first-line treatment of CRC. Unfortunately, 50% of patients undergoing surgery develop postoperative complications [3], leading to increased medical expenses, longer hospital stays, and delay of postoperative recovery [4]. Previous studies have shown that postoperative complications are an independent risk factor related to poor 5-year survival and the increased overall recurrence rate [5-7].

Enhanced recovery after surgery (ERAS) is committed to reducing surgical trauma and stress, decreasing postoperative morbidity, and accelerating postoperative recovery of patients [8-10]. ERAS guidelines emphasize the importance of early mobilization after surgery [11], which can reduce adverse outcomes such as pulmonary complications, decreased skeletal muscle strength, thromboembolism, and insulin resistance [12]. It was confirmed that ERAS was safe and effective in patients undergoing CRC surgery [13,14], and early mobilization after operation was the key to the success of ERAS [15]. However, more than half of the patients did not mobilize as recommended in the ERAS guidelines [16], which meant a reliable measurement tool was needed to not only accurately monitor the postoperative activity of patients, but also to increase their enthusiasm for mobilization. One of the possibilities is to use a wearable device [17,18].

The development of wearable devices allows objective measurements of, for example, activity steps or activity time, which can be used as a feedback tool to urge patients to achieve their intended mobilization goals [17-19]. A study found it was safe to use an activity tracker with feedback after gynecological operations and may increase patients’ physical activity [20]. Several wearable devices (such as Apple Watch, Fitbit, and Garmin) that provide combined automatic and visual feedback have been identified as effective tools for cancer survivors to raise awareness of self-activity, stimulate behavior change, and improve physical activity [21,22]. However, the clinical effect on recovery or promoting movement after operation of the wearable devices has not been sufficiently evaluated in patients with CRC. The enhanced patient monitoring (ePM)/electrophysiology (ep) pod developed by Mindray supports activity time statistics, continuous vital sign monitoring, sleep monitoring, pain scores, and trend recording, has a high accuracy of postoperative activity monitoring [23], and is suitable for related research.

The present study aims to determine whether the activity incentive feedback based on a wearable device (ePM/ep pod) could reduce postoperative complications of patients with CRC within 30 days after their operations. We hypothesized that postoperative complications would be reduced in the activity feedback group compared to the usual postoperative care group.

## Methods

### Study Design and Patients

It was an open-label, evaluator-blind, randomized controlled study with 2 parallel groups.

Patients aged ≥18 years and scheduled for CRC surgery were included. The exclusion criteria were patients who (1) had American Society of Anesthesiologists (ASA) grade >III, (2) had preoperative immobility or inability to walk unaided affected by neural, muscular, or skeletal diseases, (3) had participated in other clinical studies in the past 3 months, (4) had planned reoperation within 30 days after the initial operation, and (5) had expected to be transferred to the intensive care unit after their operations.

### Ethical Considerations

The Ethics Committee of the First Affiliated Hospital of the Air Force Military Medical University approved this randomized controlled trial (approval no. KY20222299-F-1), and the study protocol was registered with the Chinese Clinical Trial Registry (ChiCTR2300068107) on July 2, 2023. All procedures complied with the Declaration of Helsinki and institutional regulations (The Ethics Committee of the First Affiliated Hospital of the Air Force Military Medical University, Medical Ethics Committee Charter) [24]. Written informed consent was obtained from all participants prior to enrollment, which included detailed explanation of the study and their right to withdraw at any time. Upon registration, each patient was assigned a unique study number, and all subsequent data analyses were performed using a deidentified dataset to ensure participant privacy and confidentiality. No financial compensation was provided for participation.

### Interventions

All patients in the trial were randomly assigned (1:1) to a feedback group or a control group. Both groups were set the same target activity time postoperatively and thus performed activities for 2 hours on the first day after operation (POD1), 4 hours on the second day after operation (POD2), and 6 hours on the third day after operation (POD3), according to the ERAS guidelines and our ERAS pathway for CRC [10]. All patients were fitted with a wireless wearable device that continuously recorded their daily ambulation durations (ePM 12M monitor placed at the bedside or ep pod monitor placed on their wrist or blood pressure pod monitor placed on their upper arm).

In the feedback group, patients received real-time visual feedback of the movement time every day through the display of the ePM. According to the wards’ routine care based on ERAS guidelines, patients were reminded to engage in activities combined with ePM/ep pod feedback to meet predefined daily step goals and also informed about the gap between the active time and the target time.

In the control group, patients’ movement time was set to not appear on the display of the ePM. Patients were not given feedback, and they were only encouraged to carry out activities according to the current care standards based on ERAS guidelines.

### Prevention and Handling of Adverse Events

If the following incidents occur, activity supervision should be suspended immediately: participants experience pain intensity >4 (from 0 to -10), electrocardiogram abnormalities (signs of arrhythmia or acute ischemia), indoor air oxygen saturation <88%, systolic blood pressure <90 mmHg, symptoms of standing intolerance (ie, dizziness, nausea, and blurred vision), exercise weakness (ie, difficulty standing), etc. In the next scheduled mobilization, if the participant is considered to be in stable physical condition after clinical evaluation, a new mobilization attempt will be conducted. If serious adverse events such as falling out of bed or falling occur, in addition to suspending activity supervision, the competent physician should also be asked to evaluate the patient’s injury and terminate the study if necessary. The researcher should immediately fill out the serious adverse event report and report it to the hospital ethics committee.

### Randomization and Blinding

Patients were randomized using block random groups (sized blocks of 2 and 4) generated by specialized staff. All random assignments were made and sealed to an envelope by intervention staff using Statistical Analysis Software. The randomization process was performed by an independent anesthesiologist at the end of the surgical procedure, separated from the ward’s nursing staff and outcome assessors to reduce potential bias.

It was impractical to blind the intervention staff in view of the intervening measure, but contrary to the outcome evaluators. To ensure blinding during outcome assessments in the surgical ward, a schedule was established to stagger the intervention and evaluation implementation. Ward staff were informed about the activity monitoring study but were not provided with any specific hypothetical details to prevent potential bias from influencing the study results. Given the intervention characteristics, blinding the patients was also deemed to be unfeasible. To minimize performance bias, patients were informed that we were evaluating 2 mobilization schemes on the understanding that neither scheme was expected to confer any potential advantage.

### Data Collection

Preoperative baseline data were collected before an operation as follows: demographic data; preoperative medication and abdominal surgery history, and preoperative radiotherapy and chemotherapy history; vital signs; available preoperative examinations; and preoperative physical activity ability assessed by the International Physical Activity Questionnaire (IPAQ) [25]. Operation-related data collected on the day of surgery included surgical duration, intraoperative bleeding, site, type, and catheter placement status. Postoperative data were collected after each operation as follows: complications according to the Clavien-Dindo classification during hospitalization and 1 week, 2 weeks, and 1 month after the operation; the wearable device ep pod record of the activity time; digital pain Numeric Rating Scale (NRS) scores of rest and exercise; length of hospitalization; time of first exhaust and defecation; and opioids consumption; when discharged from hospital, patients in the intervention group were asked about concerns for feedback on activity time and whether they would supplement activities.

### Outcome Measurement

#### Primary Outcome

The primary outcome was the comprehensive complication index (CCI) [26] within 30 days after surgery. The CCI measured the overall complication calculated for each patient, ranging from 0 (no complication) to 100 (death), including any minor complications instead of focusing on specific ones. It was thus an objective measure with comprehensive and accurate evaluation [27-29]. When calculating CCI, all postoperative complications were evaluated and classified based on the Clavien-Dindo classification, then converted into a weighted continuous scale including quantity and severity. Finally, the CCI value was evaluated using the CCI calculator available [30].

#### Secondary Outcomes

Daily activity time was set from 12 AM to 11:59 PM based on the data uploaded by ePM/ep pod until the 4th day or until discharge, whichever came first. The patient’s compliance was considered to be low [31] on the condition that the patient wore it for less than 8 hours between 8 AM and 8 PM. The degree of pain for rest and exercise in the first 3 days after operation was scored by the NRS [32]. NRS used 0‐10 points to represent different degrees of pain scored by patients according to their subjective feelings, 0 for no pain and 10 for severe pain. The length of stay was set as the actual length of stay after a patient’s operation. The percentage of reaching the scheduled mobilization target was calculated. The patient’s activity was considered to be not up to standard if the daily activity time after their operation did not reach the target time. The mortality rate of 30 days after operation was also counted. The first exhaust and defecating times were subject to the patient’s own assessment, meaning that an obvious feeling of exhaust or defecation was reported.

### Sample Size Calculation

The sample size calculation was based on the primary outcome measured by the CCI 30 days after operation. CCI represents the measurement data that follows a normal distribution, as detailed in the previous trials on postoperative complications [33,34]. The average postoperative CCI of CRC was 10 (SD 5). Assuming that the visual feedback of the movement time could reduce CCI by 25%, the average CCI of the feedback group was set to 7.5 (SD 5) [35]. Based on the above effect size, Power Analysis and Sample Size 2015 Software was used to calculate that a minimum of 172 patients (n=86 per group) would provide 90% power to detect a 25% decrease of CCI and an α of .05 (2-sided test), assuming the feedback group was more favorable compared to the control group [35]. To compensate for a potential 20% dropout rate, 216 patients were required to be included (n=108 per group).

### Statistical Analysis

We followed the intention-to-treat principle analyzing all random patients in the baseline analysis. The analysis results of the study were based on the modified intention-to-treat (MITT) set. Statistical analyses were performed using SPSS version 23.0. The 2-sided significance level was set at a *P* value <.05.

Continuous variables with independent access to mean and SD were initially subjected to the Kolmogorov-Smirnov test to assess their normality. Otherwise, the Shapiro-Wilk test was chosen. The Levene test was used for the analysis of homogeneity of variance. Data with skewed distributions are reported as medians with IQR, while data adhering to a normal distribution are given as means with SD. Categorical variables are expressed as frequencies, percentages, and ratios. For baseline comparisons, normally distributed continuous variables with comparable variance were analyzed using Student *t* test. In contrast, continuous variables with skewed distributions or uneven variance were analyzed with the Mann-Whitney *U* test. Categorical variables were compared using either the chi-square or Fisher exact tests.

The impact of the primary outcome CCI within 30 days after an operation was analyzed using the Student *t* test or the Mann-Whitney *U* test. Except for the MITT analysis, subjects with low device compliance (wearing ePM/ep pod for less than 8 h from 8 AM to 8 PM) were excluded for sensitivity analysis of the primary outcome. In addition, subgroup analyses (preselected age, ASA, preoperative chemoradiotherapy, surgical site, and preoperative mobility) were carried out. Simultaneously, we examined the interaction between the treatment effect and these 5 subgroup variables. In addition, we statistically described the distribution of postoperative complications at all levels in the 2 groups and performed a chi-square test between the groups. As a post hoc analysis, the Spearman/point-biserial correlation coefficient was used to determine the correlation between CCI and the postoperative movement time. In the preliminary analysis, the linear regression model showed a good fitting effect. Based on this result, we conducted a linear regression analysis to assess the relationship between the movement time of POD3 (for patients discharged on POD3, the activity time on POD2 was used to fill in the data) and CCI, adjusted for potential factors such as age, ASA, sex, preoperative chemoradiotherapy, and BMI, all factors which could influence postoperative recovery.

In analyses of secondary outcomes, the Mann-Whitney *U* or Student *t* tests were used to analyze the other measurement data except for repeated measurements. The chi-square test was used to analyze the percentage of patients who achieved the predetermined mobilization goal. Binary logistic regression analysis was used to determine the determinants of patient cohorts to achieve mobilization goals. The regression model considered the following variables: age, sex, ASA grade, surgical site, and the IPAQ [25]. Repeated measurement of the NRS pain score, using a generalized linear mixed model with multinomial distribution, was used for within-group comparisons.

## Results

**Figure 1.**
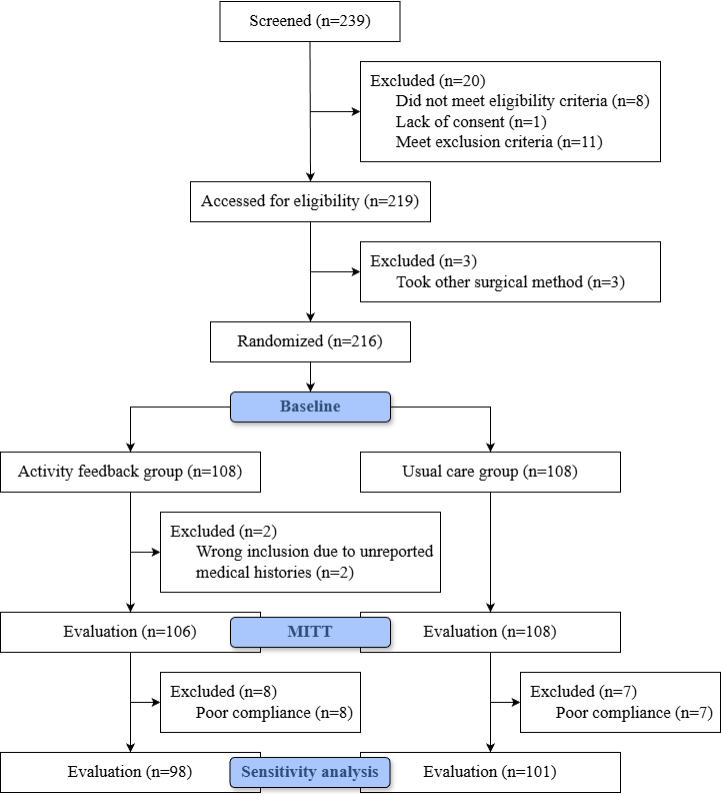
Flowchart showing patient inclusion and exclusion.

A total of CRC 239 patients were recruited between February 2023 and September 2023. Of these, 216 were randomized, 108 into usual care and 108 with activity feedback. Two patients who met the exclusion criteria were inadvertently enrolled due to unreported medical histories and were subsequently excluded from the MITT analysis (Figure 1). Patients in each randomized group had similar baseline and perioperative characteristics (Table 1).

**Figure 2.**
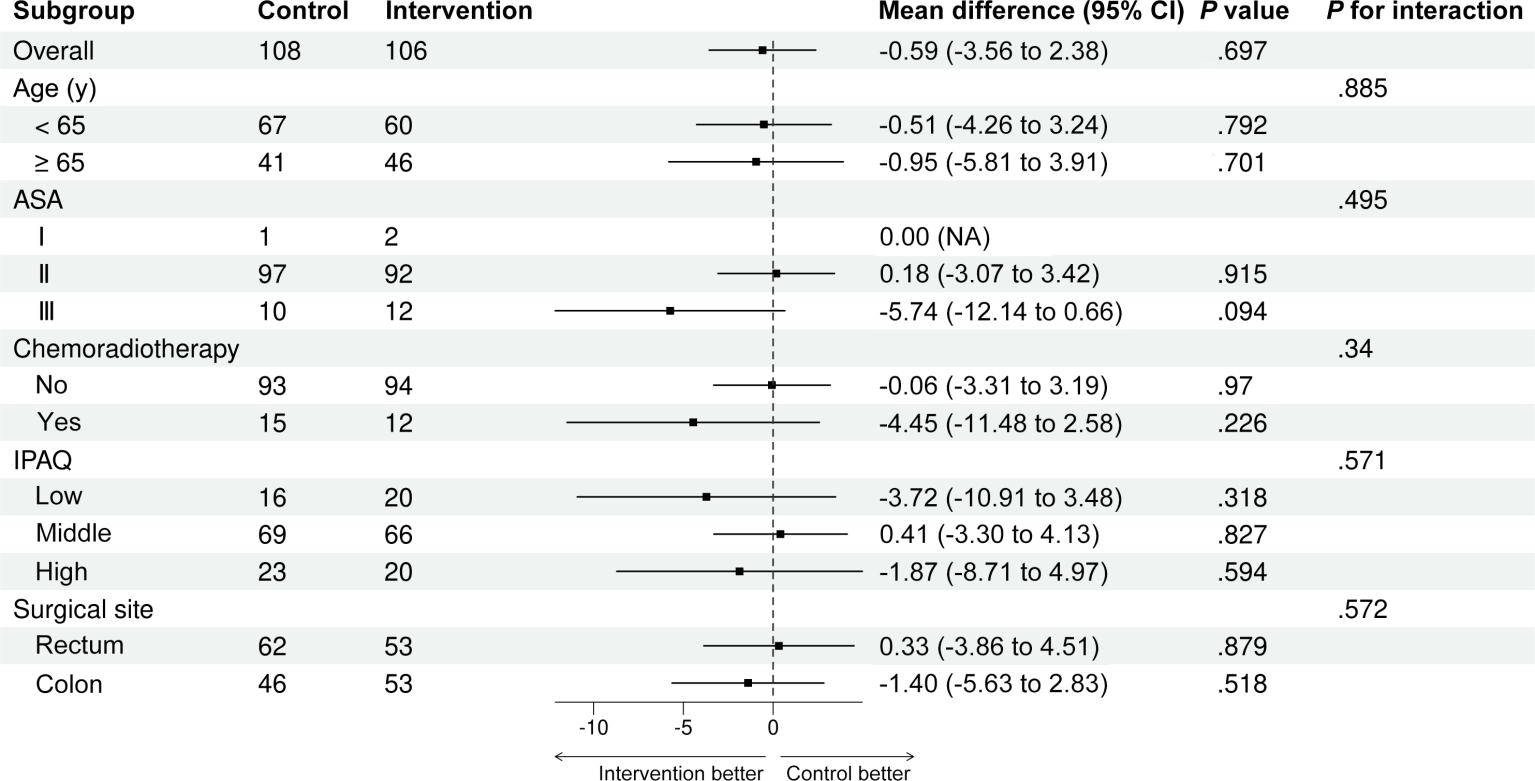
Subgroup analysis forest plot for primary outcome indicators. ASA: American Society of Anesthesiologists; IPAQ: International Physical Activity Questionnaire; NA: not available.

As shown in Table 2, no significant differences were found in CCI within 30 postoperative days between the control group, with a median CCI of 0 (range, 0‐20.90), and the activity feedback group, with a median CCI of 0 (range, 0‐12.20). The estimated mean difference between the 2 groups was −0.59 (95% CI −3.56 to 2.38; *P*=.66). Removing 15 patients with low device compliance as a sensitivity analysis, the estimated mean difference was −0.98 (95% CI −4.07 to 2.10; *P*=.54). The subgroup analysis results revealed no significant interaction between the two groups on CCI within 30 days and all the subgroup variables (Figure 2 and Checklist 1).

**Table 1.** Baseline and perioperative characteristics.

Patient characteristics	Usual care (n=108)	Activity feedback (n=108)	Mann-Whitney *U* test	Chi-square (*df*)	*t* test (*df*)	ρ	Continuity correction factor	*P* value
Age (y)	60.5 (54.25‐68.75)	63 (55-68)	5998.5	—	—	—	—	.72
Sex, n (%)			—	0.020 (1)	—	—	—	.89
Male	67 (62)	68 (62)						
Female	41 (38)	40 (38)						
BMI^a^ (kg/m²), mean (SD)	23.5 (3.3)	23.6 (3.2)	—	—	0.125 (214)	—	—	.90
ASA^b^ grade, n (%)			—	—	—	0.016	—	.82
I	1 (0.9)	2 (1.9)						
II	97 (89.8)	94 (87.0)						
III	10 (9.3)	12 (11.1)						
Smoking history, n (%)	22 (20.4)	19 (17.6)	—	0.271 (1)	—	—	—	.60
POC^c^, n (%)	46 (42.6)	44 (40.7)	—	0.076 (1)	—	—	—	.78
Hypertension, n (%)	34 (31.5)	33 (30.6)	—	0.022 (1)	—	—	—	.88
Diabetes, n (%)	20 (18.5)	12 (11.1)	—	2.348 (1)	—	—	—	.13
CHD^d^, n (%)	4 (3.7)	9 (8.3)	—	2.046 (1)	—	—	—	.15
Preoperative chemoradiotherapy, n (%)	15 (13.9)	12 (11.1)	—	0.381 (1)	—	—	—	.54
IPAQ^e^, n (%)			—	—	—	0.053	—	.43
High	23 (21.3)	20 (18.5)						
Medium	69 (63.9)	68 (63.0)						
Low	16 (14.8)	20 (18.5)						
Surgical site, n (%)			—	1.506 (1)	—	—	—	.22
Colon	46 (42.6)	55 (50.9)						
Rectum	62 (57.4)	53 (49.1)						
Laparoscopic, n (%)	103 (95.4)	105 (97.2%)	—	—	—	—	0.130	.72
Converted, n (%)	1 (0.9)	0	—	—	—	—	0.000	>.99
Surgery duration (min), median (IQR)	150 (125‐183.75)	155 (130-185)	6031.5	—	—	—	—	.66
Bleeding volume (ml), median (IQR)	50 (20-50)	50 (20-50)	5989.5	—	—	—	—	.71
POEM^f^ (mg), mean (SD)	56.3 (27.9)	58.1 (31.3)	—	—	0.446 (214)	—	—	.66

a BMI: Body Mass Index.

b ASA: American Society of Anesthesiologists.

c POC: preoperative complications.

d CHD: coronary heart disease.

e IPAQ: International Physical Activity Questionnaire.

f POEM: postoperative equivalent morphine consumption. Sufentanil 0.01 mg equal to morphine 10 mg; Hydromorphone 1.5 mg equal to morphine 10 mg; Nalbuphine 10 mg equal to morphine 10 mg [36].

**Table 2.** Between-group difference in CCI^a^ 30 days after operation.

	Usual care (n=108)	Activity feedback (n=106)	Mean difference	*P* value
CCI, median (IQR)	0 (0 to 20.90)	0 (0 to 12.20)	−0.59 (−3.56 to 2.38)	.66
CCI^b^, median (IQR)	0 (0 to 20.90)	0 (0 to 14.38)	−0.98 (−4.07 to 2.10)	.54
Maximum complication level, n (%)				
0	63 (58.3)	64 (60.4)	—^c^	—
1	14 (13)	17 (16)	—	—
2	26 (24.1)	21 (19.8)	—	—
3^b^	0	1 (0.9)	—	—
3^d^	5 (4.6)	1 (0.9)	—	—
4^b^	0	1 (0.9)	—	—
4^d^	0	1 (0.9)	—	—
5 (death)	0	0	—	—
Severe complications^e^, n (%)	5 (4.6)	4 (3.8)	—	—

a CCI: comprehensive complications index.

b Removing 15 patients with low device compliance.

c Not available.

d CCI grading was according to the classification of Clavien-Dindo classification.

e Severe complication defined as complication ≥3 [35].

The rate of no complications accounted for 58.3% (63/108) of patients in the usual care group and 60.4% (64/106) in the activity feedback group, while severe complications accounted for 4.6% (5/108) and 3.8% (4/106), respectively (Table 2). Both groups had the highest proportion of grade 2 complications. In addition, the incidence of urinary retention was the highest (15/108, 13.9% in the usual care group and 11/106, 10.4% in the activity feedback group) among the operative complications. The incidence rates of complications of infectious, respiratory system, and cardiovascular systems were 6.5% (7/108) vs 9.4% (11/106), 5.5% (6/108) vs 2.8% (3/106), and 1.9% (1/108) vs 1.8% (2/106), respectively, in the usual care group and in the activity feedback group (Table 3). On the analysis of specific complications, by the Fisher exact test, the incidence rate for pneumonia in the routine care group was 4.6% (5/108) and that in the activity feedback group was 1.9% (2/106), *P*=.45; the incidence rate for ileus in the routine care group was 0.9% (1/108) and that in the activity feedback group was 3.8% (4/106), *P*=.21, which indicated that the intervention had no significant effect on the incidence of these specific complications.

**Table 3.** Type of complications 30 days after operation.

	Usual care (n=108), n (%)	Activity feedback (n=106), n (%)
Cardiovascular system		
Acute myocardial infarction	0 (0)	1 (0.9)
Arrhythmia	2 (1.9)	1 (0.9)
Respiratory system		
Pneumonia	5 (4.6)	2 (1.9)
Atelectasis	1 (0.9)	0 (0)
Pleural effusion	0	1 (0.9)
Infectious complications		
Wound infection	3 (2.8)	6 (5.7)
Intraperitoneal or retroperitoneal abscess	0	2 (1.9)
Other infections^a^	4 (3.7)	3 (2.8)
Operative complication		
Anastomotic leakage	6 (5.6)	5 (4.7)
Ileus	1 (0.9)	4 (3.8)
Hemorrhage	0 (0)	3 (2.8)
Anemia	8 (7.4)	4 (3.8)
Urinary retention	15 (13.9)	11 (10.4)
Fat liquefaction	3 (2.8)	2 (1.9)
Other gastrointestinal complications^b^	6 (5.6)	7 (6.6)

a Other infections: including leukocytosis (white blood cells) >12 and temperature >38°C, shingles.

b Other gastrointestinal complications, including diarrhea, constipation, and gastroparesis.

As shown in Table 4, the 2 groups had no significant difference in daily activity time. Similarly, there was no difference in the rate of reaching the target between groups. The usual care and activity feedback groups demonstrated no significant difference in the length of postoperative hospital stay, with a median of 4 days for both groups (*P*=.99), in the first exhaust time (37.13, IQR 22.73-58.77 h vs 38.29, IQR 22.15-57.49 h, respectively, in the usual care group and in the activity feedback group; *P*=.89), and in the first defecating time (44.50, IQR 24.04-68.88 h vs 44.13, IQR 25.81-67.44 h; *P*=.91, respectively, in the usual care group and in the activity feedback group; Table 5). The results of the generalized linear mixed model (Table 6) revealed that the NRS scores of rest and activity showed a declining trend over time, with a significant time effect that was independent of the patient groups (*P*<.001). Moreover, there were no significant interactions between the intervention and the NRS scores at rest (*P*=.54) or during movement (*P*=.89).

**Figure 3.**
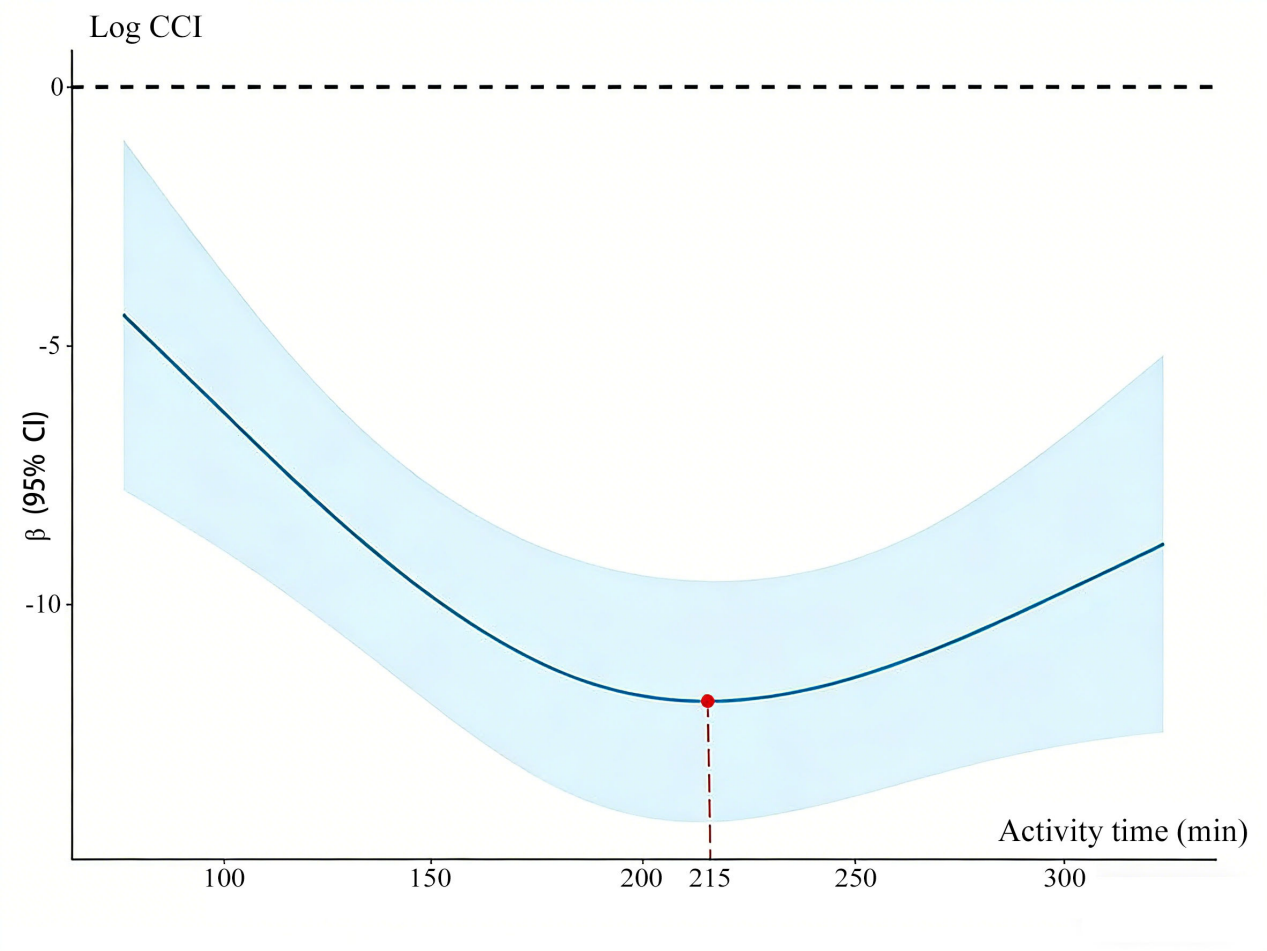
Multivariate restricted cubic spline analysis of activity time and comprehensive complications index (CCI).

In post hoc analysis, there was a significant negative correlation between CCI within the 30th postoperative day and the activity duration on POD2 (*r*=−0.166; *P*=.02) and POD3 (*r*=−0.264; *P*=.002). A linear regression analysis showed that the movement time of POD3 was negatively correlated with CCI within the 30th postoperative day, and the standardized β was −.025 (95% CI −.045 to −.006; *P*=.01). Additionally, the longer the activity time of POD3 before 215 minutes, the faster the CCI decreased; however, beyond 215 minutes, the rate of the decline slowed (Figure 3).

**Table 4.** Daily activity time and the rate of reaching the target.

	Usual care	Activity feedback	Mean difference/odds ratio (95% CI)	*P* value
Activity time, min				
POD1^a^, median (IQR)	105 (74 to 133) (n=107)	114 (65 to 131) (n=105)	−0.7 (−13.4 to 12.0)^b^	.89
POD2^c^, median (IQR)	174 (117 to 225) (n=103)	183 (134.5 to 229.5) (n=101)	5.2 (−13.1 to 23.5)^b^	.58
POD3^d^, median (IQR)	201 (132 to 248) (n=71)	195 (125.8 to 260.5) (n=70)	−3.8 (−31.8 to 24.1)^b^	.73
Reaching the target				
POD1, n/N (%)	40/107 (37.4)	46/105 (43.8)	0.8 (0.4 to 1.4)^e^	.34
POD2, n/N (%)	20/103 (19.4)	21/101 (20.8)	1.1 (0.5 to 2.2)^e^	.81
POD3, n/N (%)	4/71 (5.6)	2/70 (2.9)	0.5 (0.1 to 3.0)^e^	.69

a POD1: the first day after operation.

b Mean difference (95% CI).

c POD2: the second day after operation.

d POD3: the third day after operation.

e Odds ratio (95% CI).

**Table 5.** Postoperative recovery index.

	Usual care (n=108), median (IQR)	Activity feedback (n=106), median (IQR)	Mean difference (95% CI)	*P* value
LOS^a^ (d)	4 (3 to 5)	4 (4 to 5)	−0.4 (−1.49 to 0.69)	.47
First exhaust time (h)	37.13 (22.73 to 58.77)	38.29 (22.15 to 57.49)	0.13 (−8.07 to 8.34)	.98
First defecating time (h)	44.50 (24.04 to 68.88)	45.13 (25.81 to 67.44)	−2.99 (−14.00 to 8.03)	.60

a LOS: length of postoperative hospital stay.

**Table 6.** Pain scores after surgery at rest or movement.

Pain scores	Usual care (n=108), median (IQR)	Activity feedback (n=106), median (IQR)	Fixed effect *P* value		
			Group	Time	Group*time
NRS^a^ at rest			.95	.007	.54
POD1^b^	1 (0‐2)	1 (0‐2)			
POD2^c^	0 (0‐2)	0 (0‐2)			
POD3^d^	1 (0‐1.75)	1 (0‐1)			
NRS at movement			.32	.001	.89
POD1	2 (1‐4)	2 (2‐4)			
POD2	2 (1‐3.75)	2 (1‐4)			
POD3	2.5 (2‐3)	2.5 (1.75‐3)			

a NRS: Numeric Rating Scale.

b POD1: the first day after operation.

c POD2: the second day after operation.

d POD3: the third day after operation.

## Discussion

### Principal Findings

In this study, the activity incentive feedback based on a wearable device (ePM/ep pod) did not reduce the CCI of patients with CRC within postoperative 30 days. The differences between groups in terms of daily activity time, length of postoperative hospital stay, intestinal function recovery, and NRS pain scores were also not significant.

There may be the following reasons for the lack of difference in CCI and daily activity durations between the 2 groups. First, this study was conducted in a surgical ward with relatively deepening ERAS guidelines, where preoperative education and routine activity supervision may have facilitated effective postoperative activities even in the absence of the feedback from wearable devices. Second, all the subjects in the present study received the same activity encouragement by medical staff according to the routine care and were set the same daily activity target; therefore, patients in the control group with high compliance might obtain activity feedback through other means. Third, laparoscopy accounted for more than 95% of CRC surgery in the present study, which was similar to the type of surgery used in another study that yielded negative results regarding the hypothesis that early postoperative activity could reduce complications [15]. Future studies should investigate whether such incentive feedback would be effective for patients experiencing more intense surgical stimulation or possessing less ERAS education. Previous studies using step count feedback for mobility-enhancing showed conflicting

The assessment of early postoperative activity achievement in patients with CRC directly reflects the practical outcomes of the intervention strategies employed in this study. The activity compliance rate of patients with CRC on POD1 was 37.4% (in the usual care group) and 43.8% (in the activity monitoring feedback group), which was much lower than expected, indicating that even with the feedback mechanism, there was only a marginal improvement in patients’ adherence to the activity targets. Based on the current 6 hours recommended by ERAS guidelines, this study set a less active target of 2 hours for POD 0. In addition, the maximum activity time on POD1 was 310 minutes, which was much lower than the 6 hours recommended by ERAS guidelines. The relatively low compliance rates suggest that factors beyond the provision of activity feedback may be at play in influencing patients’ postoperative behavior, such as the intensity and appropriateness of the activity targets set. The insights gained from analyzing the achievement of early postoperative activity not only provide a deeper understanding of the limitations of the feedback intervention but also highlight the need for more tailored and patient-centered approaches to enhance postoperative mobilization. The postoperative activity time was measured by an electronic parameter objectively recorded by a wearable device, and the measurement method may be different from the considerations of previous guidelines. This may be the reason why the postoperative activity time was inconsistent with most previous studies.

Previous studies [37,38] have found patients undergoing major visceral (abdominal) surgery may not be able to achieve the mobilization goals in the early postoperative period. An observational pilot study [39] pointed out that only 50% of patients undergoing major visceral surgery could walk until POD2. The cumulative duration of postoperative activity per day ranged from 15 to 155 minutes, and only 40% of patients were satisfied with activity or physical exercise. There is a discrepancy between ERAS goals and clinical practice. Another study [40] reported that only a few patients (23.5%) achieved the mobilization goal of 6 hours (ERAS guidelines) of activity on the first day after CRC surgery. Ramírez et al [41] set the early activity goal for patients with CRC as sitting for at least 6 hours on the first postoperative day. However, the achievement rate was only 44.6%. In general, the mobilization goals described in most studies were either unclearly defined, imprecisely measured, or inaccurately monitored.

In addition, the early activity goals in ERAS did not differentiate between disease types, and radical abdominal tumor surgery is far more invasive than other simple abdominal organ partial removal. Patients with tumors were often elderly and had comorbidities, which can limit physical fitness and motivation. A study by Reed et al [42] on activity tracking after bariatric surgery found a significant negative correlation between age and the steps on POD1. Our study presented similar findings in that age was an independent factor affecting the achievement of activity on POD1. The older the patients were, the lower the possibility of reaching the activity goal. Furthermore, age negatively affected activity duration on POD2 and POD3. All patients with CRC in the present study were 61 years on average, and 42% had preoperative comorbidities, which may also be the reason for the low level of postoperative activity. Also, regression analysis showed that the activity duration of patients with colon cancer on POD2 was significantly longer than that of patients with rectal cancer, being 26.42 minutes longer. Patients with colon cancer were 2.28 times more likely than those with rectal cancer to meet the activity standard on POD2. It can be seen that the mobility of patients with colon cancer on POD2 was higher than that of patients with rectal cancer. We speculate that the reason for this finding may be that colon cancer basically does not receive radiotherapy, the proportion of preoperative chemotherapy may be lower than for rectal cancer, and the proportion of temporary ileostomy of colon cancer being much lower than that for rectal cancer. Our finding also suggests the target of early activity recommended by current ERAS guidelines (6 h in POD1) may be too high for these patients. A further increase in activity did not increase the benefit when activity levels reached a certain level. Considering our result of the regression analysis, 215 minutes might be considered as the target for activity time on POD3 to decrease CCI in patients with CRC.

In addition, several studies [43-45] have reported that early postoperative mobilization (within 24 h) was associated with fewer postoperative complications, fewer major complications, faster intestinal function recovery, and shorter hospital stays. The regression analysis in this study found that patients with high levels of physical activity before surgery were about 27 minutes more active the next day compared to patients with moderate levels of physical activity before surgery, and about 37 minutes more active than patients with low levels of physical activity before surgery. Therefore, perhaps by exploring the extent of postoperative activity decline caused by different surgical trauma, the approximate postoperative activity amount of the patient can be inferred and then combined with the preoperative activity level of the patient, a personalized postoperative activity plan can be formulated.

### Limitations

This study had some limitations. First, the 216 patients were all admitted to one hospital; therefore, the conclusion still needs to be verified in more hospitals where ERAS guidelines are less practiced. The low proportion of ASA 3 patients in this study may limit our comprehensive assessment of the benefits of patients with high comorbidities from feedback from postoperative activities. Future research may consider including more ASA3 patients to better understand the response of this special population to postoperative activity feedback intervention. Second, since it was not possible to blind the patients in this study, the 2 groups of patients may have influenced each other. Due to practical difficulties such as high patient mobility and limited follow-up time, this study failed to collect information about the views of patients and clinicians with the wearable device and how motivated they felt by wearing this. In the future, the study can be expanded to multiple wards to achieve cluster randomization, which may reduce bias. Future research can also be combined with qualitative items to provide a more comprehensive understanding of patient and clinician experiences and perceptions of wearable devices. Third, as 96% of patients in this study underwent laparoscopic surgery, the results may not be generalized to open surgery or other types of surgery. Although our inclusion criteria were broad, we excluded patients who had difficulty with preoperative activity or were in a poor physical condition. These patients may benefit from the incentive of activity feedback. A final limitation was there may be a difference between self-reported preoperative mobility assessed by IPAQ, which was not designed for hospital patients, but for assessing physical activity in daily life, and actual quantitative mobility.

### Conclusions

In the context of ERAS, this randomized controlled trial did not find evidence that activity stimulation based on feedback from ePM/ep pod wearable devices could reduce 30-day postoperative CCI in patients who underwent CRC surgery. However, ePM/ep pod could accurately record daily activity duration, which may be negatively correlated with CCI on POD3.

## Supplementary material

10.2196/70534 Checklist 1 CONSORT-eHEALTH (V 1.6.1) checklist [46].

## Abbreviations

ASA: American Society of Anesthesiologists
CCI: comprehensive complications index
CRC: colorectal cancer
ep pod: electrophysiology pod
ePM: enhanced patient monitoring
ERAS: enhanced recovery after surgery
IPAQ: International Physical Activity Questionnaire
MITT: modified intention-to-treat
NRS: Numeric Rating Scale
POD1: the first day after operation
POD2: the second day after operation
POD3: the third day after operation
