# Supplementary material for: Impact of Mobilization Facilitated by Wearable Device Enhanced Patient Monitoring/Electrophysiology Pod–Based Feedback on Postoperative Complications Following Colorectal Cancer Surgery: Randomized Controlled Trial
Source: JMIR Mhealth Uhealth. 2026 Jan 30;14:e70534. doi: 10.2196/70534 (PMC12858115; doi:10.2196/70534)
Supplement: Checklist 1 [file mhealth-v14-e70534-s001.pdf]

CONSORT-EHEALTH (V 1.6.1) - Submission/Publication Form

The CONSORT-EHEALTH checklist is intended for authors of randomized trials evaluating web-based and Internet-based applications/interventions, including mobile interventions, electronic games (incl multiplayer games), social media, certain telehealth applications, and other interactive and/or networked electronic applications. Some of the items (e.g. all subitems under item 5 - description of the intervention) may also be applicable for other study designs.

The goal of the CONSORT EHEALTH checklist and guideline is to be
a) a guide for reporting for authors of RCTs,
b) to form a basis for appraisal of an ehealth trial (in terms of validity)

CONSORT-EHEALTH items/subitems are MANDATORY reporting items for studies published in the Journal of Medical Internet Research and other journals / scientific societies endorsing the checklist.

Items numbered 1., 2., 3., 4a., 4b etc are original CONSORT or CONSORT-NPT (non-pharmacologic treatment) items.
Items with Roman numerals (i., ii, iii, iv etc.) are CONSORT-EHEALTH extensions/clarifications.

As the CONSORT-EHEALTH checklist is still considered in a formative stage, we would ask that you also RATE ON A SCALE OF 1-5 how important/useful you feel each item is FOR THE PURPOSE OF THE CHECKLIST and reporting guideline (optional).

Mandatory reporting items are marked with a red *.
In the textboxes, either copy & paste the relevant sections from your manuscript into this form - please include any quotes from your manuscript in QUOTATION MARKS,
or answer directly by providing additional information not in the manuscript, or elaborating on why the item was not relevant for this study.

YOUR ANSWERS WILL BE PUBLISHED AS A SUPPLEMENTARY FILE TO YOUR PUBLICATION IN JMIR AND ARE CONSIDERED PART OF YOUR PUBLICATION (IF ACCEPTED).
Please fill in these questions diligently. Information will not be copyedited, so please use proper spelling and grammar, use correct capitalization, and avoid abbreviations.

DO NOT FORGET TO SAVE AS PDF _AND_ CLICK THE SUBMIT BUTTON SO YOUR ANSWERS ARE IN OUR DATABASE !!!

Citation Suggestion (if you append the pdf as Appendix we suggest to cite this paper in the caption):
Eysenbach G, CONSORT-EHEALTH Group
CONSORT-EHEALTH: Improving and Standardizing Evaluation Reports of Web-based and Mobile Health Interventions
J Med Internet Res 2011;13(4):e126

URL: http://www.jmir.org/2011/4/e126/
doi: 10.2196/jmir.1923
PMID: 22209829

mw250821701@gmail.com 切换账号

未共享的内容

重新提交才能保存

* 表示必填

Your name *

First Last

Huang Nie

Primary Affiliation (short), City, Country *

University of Toronto, Toronto, Canada

The Fourth Military Medical University Xijing H

Your e-mail address *

abc@gmail.com

niehuang@163.com

Title of your manuscript *

Provide the (draft) title of your manuscript.

Impact of Mobilization Facilitated by Wearable Device ePM/ep Pod-based Feedback on Postoperative Complications following Colorectal Cancer Surgery: Randomized Controlled Trial

Name of your App/Software/Intervention *

If there is a short and a long/alternate name, write the short name first and add the long name in brackets.

Wearable Device ePM/ep Pod

Evaluated Version (if any)

e.g. "V1", "Release 2017-03-01", "Version 2.0.27913"

您的回答

Language(s) *

What language is the intervention/app in? If multiple languages are available, separate by comma (e.g. "English, French")

English

URL of your Intervention Website or App

e.g. a direct link to the mobile app on app in appstore (itunes, Google Play), or URL of the website. If the intervention is a DVD or hardware, you can also link to an Amazon page.

您的回答

URL of an image/screenshot (optional)

您的回答

Accessibility *

Can an enduser access the intervention presently?

- ( ) access is free and open
- ( ) access only for special usergroups, not open
- (•) access is open to everyone, but requires payment/subscription/in-app purchases
- ( ) app/intervention no longer accessible
- ( ) 其他:

Primary Medical Indication/Disease/Condition *

e.g. "Stress", "Diabetes", or define the target group in brackets after the condition, e.g. "Autism (Parents of children with)", "Alzheimers (Informal Caregivers of)"

Colorectal cancer (after operation)

Primary Outcomes measured in trial *

comma-separated list of primary outcomes reported in the trial

The Comprehensive Complication Index (CCI)

Secondary/other outcomes

Are there any other outcomes the intervention is expected to affect?

Secondary outcomes included daily activity time, pain Numeric Rating Scale (NRS) scores for rest and movement during the first three postoperative days, length of stay, percentage of reaching the scheduled mobilization target, 30-day postoperative mortality rate, and the times of first exhaust and defecation.

Recommended "Dose" *

What do the instructions for users say on how often the app should be used?

- ◯ Approximately Daily
- ◯ Approximately Weekly
- ◯ Approximately Monthly
- ◯ Approximately Yearly
- ◯ "as needed"
- ◉ 其他: Continuously used during the first three-day postoperative

Approx. Percentage of Users (starters) still using the app as recommended after 3 months *

- ◉ unknown / not evaluated
- ◯ 0-10%
- ◯ 11-20%
- ◯ 21-30%
- ◯ 31-40%
- ◯ 41-50%
- ◯ 51-60%
- ◯ 61-70%
- ◯ 71%-80%
- ◯ 81-90%
- ◯ 91-100%
- ◯ 其他:

Overall, was the app/intervention effective? *

- ( ) yes: all primary outcomes were significantly better in intervention group vs control
- ( ) partly: SOME primary outcomes were significantly better in intervention group vs control
- (•) no statistically significant difference between control and intervention
- ( ) potentially harmful: control was significantly better than intervention in one or more outcomes
- ( ) inconclusive: more research is needed
- ( ) 其他:

Article Preparation Status/Stage *

At which stage in your article preparation are you currently (at the time you fill in this form)

- ( ) not submitted yet - in early draft status
- ( ) not submitted yet - in late draft status, just before submission
- ( ) submitted to a journal but not reviewed yet
- ( ) submitted to a journal and after receiving initial reviewer comments
- (•) submitted to a journal and accepted, but not published yet
- ( ) published
- ( ) 其他:

Journal *

If you already know where you will submit this paper (or if it is already submitted), please provide the journal name (if it is not JMIR, provide the journal name under "other")

- ( ) not submitted yet / unclear where I will submit this
- ( ) Journal of Medical Internet Research (JMIR)
- (•) JMIR mHealth and UHealth
- ( ) JMIR Serious Games
- ( ) JMIR Mental Health
- ( ) JMIR Public Health
- ( ) JMIR Formative Research
- ( ) Other JMIR sister journal
- ( ) 其他:

Is this a full powered effectiveness trial or a pilot/feasibility trial? *

- ( ) Pilot/feasibility
- (•) Fully powered

Manuscript tracking number *

If this is a JMIR submission, please provide the manuscript tracking number under "other" (The ms tracking number can be found in the submission acknowledgement email, or when you login as author in JMIR. If the paper is already published in JMIR, then the ms tracking number is the four-digit number at the end of the DOI, to be found at the bottom of each published article in JMIR)

- ( ) no ms number (yet) / not (yet) submitted to / published in JMIR
- (•) 其他: #70534

TITLE AND ABSTRACT

1a) TITLE: Identification as a randomized trial in the title

1a) Does your paper address CONSORT item 1a? *

I.e does the title contain the phrase "Randomized Controlled Trial"? (if not, explain the reason under "other")

- (●) yes
- ( ) 其他:

1a-i) Identify the mode of delivery in the title

Identify the mode of delivery. Preferably use “web-based” and/or “mobile” and/or “electronic game” in the title. Avoid ambiguous terms like “online”, “virtual”, “interactive”. Use “Internet-based” only if Intervention includes non-web-based Internet components (e.g. email), use “computer-based” or “electronic” only if offline products are used. Use “virtual” only in the context of “virtual reality” (3-D worlds). Use “online” only in the context of “online support groups”. Complement or substitute product names with broader terms for the class of products (such as “mobile” or “smart phone” instead of “iphone”), especially if the application runs on different platforms.

	1	2	3	4	5	
subitem not at all important	( )	( )	( )	( )	(●)	essential

清除所选内容

Does your paper address subitem 1a-i? *

Copy and paste relevant sections from manuscript title (include quotes in quotation marks "like this" to indicate direct quotes from your manuscript), or elaborate on this item by providing additional information not in the ms, or briefly explain why the item is not applicable/relevant for your study

"Wearable Device ePM/ep Pod-based Feedback on Postoperative Complications"
The Mindray ePM/ep pod, capable of tracking activity, vital signs, sleep, and pain, offers high-precision postoperative monitoring and is well-suited for research on activity feedback.

1a-ii) Non-web-based components or important co-interventions in title

Mention non-web-based components or important co-interventions in title, if any (e.g., “with telephone support”).

	1	2	3	4	5	
subitem not at all important	○	○	○	○	○	essential

Does your paper address subitem 1a-ii?

Copy and paste relevant sections from manuscript title (include quotes in quotation marks "like this" to indicate direct quotes from your manuscript), or elaborate on this item by providing additional information not in the ms, or briefly explain why the item is not applicable/relevant for your study

您的回答

1a-iii) Primary condition or target group in the title

Mention primary condition or target group in the title, if any (e.g., “for children with Type I Diabetes”) Example: A Web-based and Mobile Intervention with Telephone Support for Children with Type I Diabetes: Randomized Controlled Trial

	1	2	3	4	5	
subitem not at all important	○	○	○	○	◉	essential

清除所选内容

Does your paper address subitem 1a-iii? *

Copy and paste relevant sections from manuscript title (include quotes in quotation marks "like this" to indicate direct quotes from your manuscript), or elaborate on this item by providing additional information not in the ms, or briefly explain why the item is not applicable/relevant for your study

Patients "following Colorectal Cancer Surgery"

1b) ABSTRACT: Structured summary of trial design, methods, results, and conclusions

NPT extension: Description of experimental treatment, comparator, care providers, centers, and blinding status.

1b-i) Key features/functionalities/components of the intervention and comparator in the METHODS section of the ABSTRACT

Mention key features/functionalities/components of the intervention and comparator in the abstract. If possible, also mention theories and principles used for designing the site. Keep in mind the needs of systematic reviewers and indexers by including important synonyms. (Note: Only report in the abstract what the main paper is reporting. If this information is missing from the main body of text, consider adding it)

	1	2	3	4	5	
subitem not at all important	○	○	○	○	◉	essential

清除所选内容

Does your paper address subitem 1b-i? *

Copy and paste relevant sections from the manuscript abstract (include quotes in quotation marks "like this" to indicate direct quotes from your manuscript), or elaborate on this item by providing additional information not in the ms, or briefly explain why the item is not applicable/relevant for your study

"Patients were randomized to a feedback group or a control group. Both groups were set the same target activity time postoperatively based on ERAS guidelines. The feedback group received real-time visual feedback of movement time daily through the ep-pod device, while the control group did not receive feedback."

1b-ii) Level of human involvement in the METHODS section of the ABSTRACT

Clarify the level of human involvement in the abstract, e.g., use phrases like “fully automated” vs. “therapist/nurse/care provider/physician-assisted” (mention number and expertise of providers involved, if any). (Note: Only report in the abstract what the main paper is reporting. If this information is missing from the main body of text, consider adding it)

	1	2	3	4	5	
subitem not at all important	○	○	○	○	○	essential

Does your paper address subitem 1b-ii?

Copy and paste relevant sections from the manuscript abstract (include quotes in quotation marks "like this" to indicate direct quotes from your manuscript), or elaborate on this item by providing additional information not in the ms, or briefly explain why the item is not applicable/relevant for your study

您的回答

1b-iii) Open vs. closed, web-based (self-assessment) vs. face-to-face assessments in the METHODS section of the ABSTRACT

Mention how participants were recruited (online vs. offline), e.g., from an open access website or from a clinic or a closed online user group (closed usergroup trial), and clarify if this was a purely web-based trial, or there were face-to-face components (as part of the intervention or for assessment). Clearly say if outcomes were self-assessed through questionnaires (as common in web-based trials). Note: In traditional offline trials, an open trial (open-label trial) is a type of clinical trial in which both the researchers and participants know which treatment is being administered. To avoid confusion, use “blinded” or “unblinded” to indicated the level of blinding instead of “open”, as “open” in web-based trials usually refers to “open access” (i.e. participants can self-enrol). (Note: Only report in the abstract what the main paper is reporting. If this information is missing from the main body of text, consider adding it)

	1	2	3	4	5	
subitem not at all important	○	○	○	○	○	essential

Does your paper address subitem 1b-iii?

Copy and paste relevant sections from the manuscript abstract (include quotes in quotation marks "like this" to indicate direct quotes from your manuscript), or elaborate on this item by providing additional information not in the ms, or briefly explain why the item is not applicable/relevant for your study

您的回答

1b-iv) RESULTS section in abstract must contain use data

Report number of participants enrolled/assessed in each group, the use/uptake of the intervention (e.g., attrition/adherence metrics, use over time, number of logins etc.), in addition to primary/secondary outcomes. (Note: Only report in the abstract what the main paper is reporting. If this information is missing from the main body of text, consider adding it)

	1	2	3	4	5	
subitem not at all important	○	○	○	○	◉	essential

清除所选内容

Does your paper address subitem 1b-iv?

Copy and paste relevant sections from the manuscript abstract (include quotes in quotation marks "like this" to indicate direct quotes from your manuscript), or elaborate on this item by providing additional information not in the ms, or briefly explain why the item is not applicable/relevant for your study

"239 patients were recruited between February 2023 and September 2023, with 206 randomized (108 for each group). There was no significant difference in CCI within 30 postoperative days between the control group (median CCI 0, range 0–20.90) and the activity feedback group (median CCI 0, range 0–12.20)"

1b-v) CONCLUSIONS/DISCUSSION in abstract for negative trials

Conclusions/Discussions in abstract for negative trials: Discuss the primary outcome - if the trial is negative (primary outcome not changed), and the intervention was not used, discuss whether negative results are attributable to lack of uptake and discuss reasons. (Note: Only report in the abstract what the main paper is reporting. If this information is missing from the main body of text, consider adding it)

	1	2	3	4	5	
subitem not at all important	○	○	○	○	○	essential

Does your paper address subitem 1b-v?

Copy and paste relevant sections from the manuscript abstract (include quotes in quotation marks "like this" to indicate direct quotes from your manuscript), or elaborate on this item by providing additional information not in the ms, or briefly explain why the item is not applicable/relevant for your study

您的回答

INTRODUCTION

2a) In INTRODUCTION: Scientific background and explanation of rationale

2a-i) Problem and the type of system/solution

Describe the problem and the type of system/solution that is object of the study: intended as stand-alone intervention vs. incorporated in broader health care program? Intended for a particular patient population? Goals of the intervention, e.g., being more cost-effective to other interventions, replace or complement other solutions? (Note: Details about the intervention are provided in “Methods” under 5)

	1	2	3	4	5	
subitem not at all important	○	○	○	○	○	essential

Does your paper address subitem 2a-i? *

Copy and paste relevant sections from the manuscript (include quotes in quotation marks "like this" to indicate direct quotes from your manuscript), or elaborate on this item by providing additional information not in the ms, or briefly explain why the item is not applicable/relevant for your study

"Colorectal cancer (CRC) is the second most common cancer in China and ranks third globally in mortality rate [1,2]. 50% of patients undergoing surgery develop postoperative complications [3]. Postoperative complications are an independent risk factor related to poor 5-year survival and the increased overall recurrence rate [5-7]."
"Enhanced Recovery After Surgery (ERAS) is committed to reducing surgical trauma and stress, reducing postoperative complications, and accelerating postoperative recovery of patients 8-10]. ERAS guidelines emphasize the importance of early mobilization after surgery [11]. But more than half of the patients did not mobilize as recommended in the ERAS guidelines [16], which meant a reliable measurement tool was needed."
"The clinical effect on recovery or promoting movement after operation of the wearable devices has not been sufficiently evaluated in CRC patients. The ePM/ep pod developed by Mindray supports multiple monitoring functions and is suitable for related research."

2a-ii) Scientific background, rationale: What is known about the (type of) system

Scientific background, rationale: What is known about the (type of) system that is the object of the study (be sure to discuss the use of similar systems for other conditions/diagnoses, if appropiate), motivation for the study, i.e. what are the reasons for and what is the context for this specific study, from which stakeholder viewpoint is the study performed, potential impact of findings [2]. Briefly justify the choice of the comparator.

	1	2	3	4	5	
subitem not at all important	○	○	○	○	○	essential

Does your paper address subitem 2a-ii? *

Copy and paste relevant sections from the manuscript (include quotes in quotation marks "like this" to indicate direct quotes from your manuscript), or elaborate on this item by providing additional information not in the ms, or briefly explain why the item is not applicable/relevant for your study

"Early mobilization after operation was the key to the success of ERAS [15]. However, more than half of the patients did not mobilize as recommended in the ERAS guidelines [16],which meant a reliable measurement tool was needed to not only accurately monitor the postoperative activity of patients, but also to increase their enthusiasm for mobilization.One of the possibilities is to use a wearable device [17,18]."
"The development of wearable devices allows objective measurements of, for example, activity steps or activity time, which can be used as a feedback tool to urge patients to achieve their intended mobilization goals [17-19]"
"However, the clinical effect on recovery or promoting movement after operation of the wearable devices has not been sufficiently evaluated in CRC patients. The ePM/ep pod developed by Mindray supports activity time statistics, continuous vital sign monitoring, sleep monitoring at night, pain scores and trend recording, has a high accuracy of postoperative activity monitoring [23] and is suitable for related research."

2b) In INTRODUCTION: Specific objectives or hypotheses

Does your paper address CONSORT subitem 2b? *

Copy and paste relevant sections from the manuscript (include quotes in quotation marks "like this" to indicate direct quotes from your manuscript), or elaborate on this item by providing additional information not in the ms, or briefly explain why the item is not applicable/relevant for your study

"The present study aims were to determine whether the activity incentive feedback based ona wearable device (ePM/ep pod) could reduce postoperative complications of CRC patientswithin 30 days after their operations. We hypothesized that postoperative complicationswould be reduced in the activity feedback group compared to the routine postoperative care group."

METHODS

3a) Description of trial design (such as parallel, factorial) including allocation ratio

Does your paper address CONSORT subitem 3a? *

Copy and paste relevant sections from the manuscript (include quotes in quotation marks "like this" to indicate direct quotes from your manuscript), or elaborate on this item by providing additional information not in the ms, or briefly explain why the item is not applicable/relevant for your study

"It was an open-abel, evaluator-blind, randomized controlled study with two parallel groups." Randomization. "Patients were randomized using block random groups (sized blocks of 2 and 4) generated by specialized staff. All random assignments were made and sealed to an envelope by intervention staff using SAS software. The randomization process was performed by an independent anesthesiologist at the end of the surgical procedure, separated from the ward's nursing staff and outcome assessors to reduce potential bias." Blinding."It was impractical to blind the intervention staff in view of the intervening measure, but contrary to the outcome evaluators. To ensure blinding during outcome assessments in the surgical ward, a schedule was established to stagger the intervention and evaluation implementation. Ward staff were informed about the activity monitoring study but were not provided with any specific hypothetical details to prevent potential bias from influencing the study results. Given the intervention characteristics, blinding the patients was also deemed to be unfeasible. To minimize performance bias, patients were informed that we were evaluating two mobilization schemes on the understanding that neither scheme was expected to confer any potential advantage."

3b) Important changes to methods after trial commencement (such as eligibility criteria), with reasons

Does your paper address CONSORT subitem 3b? *

Copy and paste relevant sections from the manuscript (include quotes in quotation marks "like this" to indicate direct quotes from your manuscript), or elaborate on this item by providing additional information not in the ms, or briefly explain why the item is not applicable/relevant for your study

There were no important changes to methods after trial commencement.

3b-i) Bug fixes, Downtimes, Content Changes

Bug fixes, Downtimes, Content Changes: ehealth systems are often dynamic systems. A description of changes to methods therefore also includes important changes made on the intervention or comparator during the trial (e.g., major bug fixes or changes in the functionality or content) (5-iii) and other “unexpected events” that may have influenced study design such as staff changes, system failures/downtimes, etc. [2].

	1	2	3	4	5	
subitem not at all important	○	○	○	○	○	essential

Does your paper address subitem 3b-i?

Copy and paste relevant sections from the manuscript (include quotes in quotation marks "like this" to indicate direct quotes from your manuscript), or elaborate on this item by providing additional information not in the ms, or briefly explain why the item is not applicable/relevant for your study

您的回答

4a) Eligibility criteria for participants

Does your paper address CONSORT subitem 4a? *

Copy and paste relevant sections from the manuscript (include quotes in quotation marks "like this" to indicate direct quotes from your manuscript), or elaborate on this item by providing additional information not in the ms, or briefly explain why the item is not applicable/relevant for your study

"Patients aged ≥ 18 years and scheduled for CRC surgery were included. The exclusion criteria were: 1) American Society of Anesthesiologists (ASA) grade > III; 2) Preoperative immobility or inability to walk unaided affected by neural, muscular or skeletal diseases; 3) Had participated in other clinical studies in the past 3 months; 4) Planned re-operation within 30 days after the initial operation; and 5) Expected to be transferred to the Intensive Care Unit (ICU) after their operations."

4a-i) Computer / Internet literacy

Computer / Internet literacy is often an implicit “de facto” eligibility criterion - this should be explicitly clarified.

	1	2	3	4	5	
subitem not at all important	○	○	○	○	○	essential

Does your paper address subitem 4a-i?

Copy and paste relevant sections from the manuscript (include quotes in quotation marks "like this" to indicate direct quotes from your manuscript), or elaborate on this item by providing additional information not in the ms, or briefly explain why the item is not applicable/relevant for your study

Participants did not use Computer / Internet technology. They only referred to feedback from mobile devices.

4a-ii) Open vs. closed, web-based vs. face-to-face assessments:

Open vs. closed, web-based vs. face-to-face assessments: Mention how participants were recruited (online vs. offline), e.g., from an open access website or from a clinic, and clarify if this was a purely web-based trial, or there were face-to-face components (as part of the intervention or for assessment), i.e., to what degree got the study team to know the participant. In online-only trials, clarify if participants were quasi-anonymous and whether having multiple identities was possible or whether technical or logistical measures (e.g., cookies, email confirmation, phone calls) were used to detect/prevent these.

	1	2	3	4	5	
subitem not at all important	○	○	○	○	○	essential

Does your paper address subitem 4a-ii? *

Copy and paste relevant sections from the manuscript (include quotes in quotation marks "like this" to indicate direct quotes from your manuscript), or elaborate on this item by providing additional information not in the ms, or briefly explain why the item is not applicable/relevant for your study

Participants were all patients hospitalized and underwent surgery during the trial. They were registered, admitted through routine procedures. During the trial, they had face-to-face contact with medical staff and received assessments.

4a-iii) Information giving during recruitment

Information given during recruitment. Specify how participants were briefed for recruitment and in the informed consent procedures (e.g., publish the informed consent documentation as appendix, see also item X26), as this information may have an effect on user self-selection, user expectation and may also bias results.

	1	2	3	4	5	
subitem not at all important	○	○	○	○	○	essential

Does your paper address subitem 4a-iii?

Copy and paste relevant sections from the manuscript (include quotes in quotation marks "like this" to indicate direct quotes from your manuscript), or elaborate on this item by providing additional information not in the ms, or briefly explain why the item is not applicable/relevant for your study

您的回答

4b) Settings and locations where the data were collected

Does your paper address CONSORT subitem 4b? *

Copy and paste relevant sections from the manuscript (include quotes in quotation marks "like this" to indicate direct quotes from your manuscript), or elaborate on this item by providing additional information not in the ms, or briefly explain why the item is not applicable/relevant for your study

"Preoperative baseline data were collected before an operation as follows: demographic data; preoperative medication and abdominal surgery history, and preoperative radiotherapy and chemotherapy history; vital signs; available pre-operative examinations; and pre-operative physical activity ability assessed by the International Physical Activity Questionnaire (IPAQ) [24]. Operation-related data collected on the day of surgery included: surgical duration, intraoperative bleeding, site, type, and catheter placement status. Postoperative data were collected after each operation as follows: complications according to the Clavien-Dindo classification during the hospitalization and one week, two weeks and one month after the operation; the wearable device ep pod record of the activity time; digital Pain Numeric Rating Scale (NRS) scores of rest and exercise; length of hospitalization; time of first exhaust and defecation; opioids consumption; when discharged from hospital, patients in the intervention group were asked about concerns for feedback on activity time and whether they would supplement activities."

4b-i) Report if outcomes were (self-)assessed through online questionnaires

Clearly report if outcomes were (self-)assessed through online questionnaires (as common in web-based trials) or otherwise.

	1	2	3	4	5	
subitem not at all important	○	○	○	○	○	essential

Does your paper address subitem 4b-i? *

Copy and paste relevant sections from the manuscript (include quotes in quotation marks "like this" to indicate direct quotes from your manuscript), or elaborate on this item by providing additional information not in the ms, or briefly explain why the item is not applicable/relevant for your study

The outcomes were not assessed through online quensionnaires.

4b-ii) Report how institutional affiliations are displayed

Report how institutional affiliations are displayed to potential participants [on ehealth media], as affiliations with prestigious hospitals or universities may affect volunteer rates, use, and reactions with regards to an intervention.(Not a required item – describe only if this may bias results)

	1	2	3	4	5	
subitem not at all important	○	○	○	○	○	essential

Does your paper address subitem 4b-ii?

Copy and paste relevant sections from the manuscript (include quotes in quotation marks "like this" to indicate direct quotes from your manuscript), or elaborate on this item by providing additional information not in the ms, or briefly explain why the item is not applicable/relevant for your study

您的回答

5) The interventions for each group with sufficient details to allow replication, including how and when they were actually administered

5-i) Mention names, credential, affiliations of the developers, sponsors, and owners

Mention names, credential, affiliations of the developers, sponsors, and owners [6] (if authors/evaluators are owners or developer of the software, this needs to be declared in a “Conflict of interest” section or mentioned elsewhere in the manuscript).

	1	2	3	4	5	
subitem not at all important	○	○	○	○	○	essential

Does your paper address subitem 5-i?

Copy and paste relevant sections from the manuscript (include quotes in quotation marks "like this" to indicate direct quotes from your manuscript), or elaborate on this item by providing additional information not in the ms, or briefly explain why the item is not applicable/relevant for your study

您的回答

5-ii) Describe the history/development process

Describe the history/development process of the application and previous formative evaluations (e.g., focus groups, usability testing), as these will have an impact on adoption/use rates and help with interpreting results.

	1	2	3	4	5	
subitem not at all important	○	○	○	○	○	essential

Does your paper address subitem 5-ii?

Copy and paste relevant sections from the manuscript (include quotes in quotation marks "like this" to indicate direct quotes from your manuscript), or elaborate on this item by providing additional information not in the ms, or briefly explain why the item is not applicable/relevant for your study

您的回答

5-iii) Revisions and updating

Revisions and updating. Clearly mention the date and/or version number of the application/intervention (and comparator, if applicable) evaluated, or describe whether the intervention underwent major changes during the evaluation process, or whether the development and/or content was “frozen” during the trial. Describe dynamic components such as news feeds or changing content which may have an impact on the replicability of the intervention (for unexpected events see item 3b).

	1	2	3	4	5	
subitem not at all important	○	○	○	○	○	essential

Does your paper address subitem 5-iii?

Copy and paste relevant sections from the manuscript (include quotes in quotation marks "like this" to indicate direct quotes from your manuscript), or elaborate on this item by providing additional information not in the ms, or briefly explain why the item is not applicable/relevant for your study

您的回答

5-iv) Quality assurance methods

Provide information on quality assurance methods to ensure accuracy and quality of information provided [1], if applicable.

	1	2	3	4	5	
subitem not at all important	○	○	○	○	○	essential

Does your paper address subitem 5-iv?

Copy and paste relevant sections from the manuscript (include quotes in quotation marks "like this" to indicate direct quotes from your manuscript), or elaborate on this item by providing additional information not in the ms, or briefly explain why the item is not applicable/relevant for your study

您的回答

5-v) Ensure replicability by publishing the source code, and/or providing screenshots/screen-capture video, and/or providing flowcharts of the algorithms used

Ensure replicability by publishing the source code, and/or providing screenshots/screen-capture video, and/or providing flowcharts of the algorithms used. Replicability (i.e., other researchers should in principle be able to replicate the study) is a hallmark of scientific reporting.

	1	2	3	4	5	
subitem not at all important	○	○	○	○	○	essential

Does your paper address subitem 5-v?

Copy and paste relevant sections from the manuscript (include quotes in quotation marks "like this" to indicate direct quotes from your manuscript), or elaborate on this item by providing additional information not in the ms, or briefly explain why the item is not applicable/relevant for your study

您的回答

5-vi) Digital preservation

Digital preservation: Provide the URL of the application, but as the intervention is likely to change or disappear over the course of the years; also make sure the intervention is archived (Internet Archive, webcitation.org, and/or publishing the source code or screenshots/videos alongside the article). As pages behind login screens cannot be archived, consider creating demo pages which are accessible without login.

	1	2	3	4	5	
subitem not at all important	○	○	○	○	○	essential

Does your paper address subitem 5-vi?

Copy and paste relevant sections from the manuscript (include quotes in quotation marks "like this" to indicate direct quotes from your manuscript), or elaborate on this item by providing additional information not in the ms, or briefly explain why the item is not applicable/relevant for your study

您的回答

5-vii) Access

Access: Describe how participants accessed the application, in what setting/context, if they had to pay (or were paid) or not, whether they had to be a member of specific group. If known, describe how participants obtained “access to the platform and Internet” [1]. To ensure access for editors/reviewers/readers, consider to provide a “backdoor” login account or demo mode for reviewers/readers to explore the application (also important for archiving purposes, see vi).

	1	2	3	4	5	
subitem not at all important	◯	◯	◯	◯	◯	essential

Does your paper address subitem 5-vii? *

Copy and paste relevant sections from the manuscript (include quotes in quotation marks "like this" to indicate direct quotes from your manuscript), or elaborate on this item by providing additional information not in the ms, or briefly explain why the item is not applicable/relevant for your study

"All patients were fitted with a wireless wearable device that continuously recorded their daily ambulation durations (ePM 12M monitor placed at the bedside / ep pod monitorplaced on their wrist / bp pod monitor placed on their upper arm)."
The equipment was provided by the researchers. Participants only needed to wear it.

5-viii) Mode of delivery, features/functionalities/components of the intervention and comparator, and the theoretical framework

Describe mode of delivery, features/functionalities/components of the intervention and comparator, and the theoretical framework [6] used to design them (instructional strategy [1], behaviour change techniques, persuasive features, etc., see e.g., [7, 8] for terminology). This includes an in-depth description of the content (including where it is coming from and who developed it) [1],” whether [and how] it is tailored to individual circumstances and allows users to track their progress and receive feedback” [6]. This also includes a description of communication delivery channels and – if computer-mediated communication is a component – whether communication was synchronous or asynchronous [6]. It also includes information on presentation strategies [1], including page design principles, average amount of text on pages, presence of hyperlinks to other resources, etc. [1].

	1	2	3	4	5	
subitem not at all important	◯	◯	◯	◯	◯	essential

Does your paper address subitem 5-viii? *

Copy and paste relevant sections from the manuscript (include quotes in quotation marks "like this" to indicate direct quotes from your manuscript), or elaborate on this item by providing additional information not in the ms, or briefly explain why the item is not applicable/relevant for your study

"The ePM/ep pod developed by Mindray supports activity time statistics, continuous vital sign monitoring, sleep monitoring, pain scores and trend recording, has a high accuracy of postoperative activity monitoring [23] and is suitable for related research. "
"All patients were fitted with a wireless wearable device that continuously recorded their daily ambulation durations (ePM 12M monitor placed at the bedside / ep pod monitor placed on their wrist / bp pod monitor placed on their upper arm)."
"In the feedback group, patients received real-time visual feedback of the movement time every day through the display of the ePM. According to the wards' routine care based on ERAS guidelines, patients were reminded to engage in activities combined with ePM/ep pod feedback to meet predefined daily step goals, and also informed about the gap between the active time and the target time. in the control group, patients' movement time was set to not appear on the display of the ePM. Patients were not given feedback and they were only encouraged to carry out activities according to the current care standards based on ERAS guidelines."

5-ix) Describe use parameters

Describe use parameters (e.g., intended “doses” and optimal timing for use). Clarify what instructions or recommendations were given to the user, e.g., regarding timing, frequency, heaviness of use, if any, or was the intervention used ad libitum.

	1	2	3	4	5	
subitem not at all important	○	○	○	○	○	essential

Does your paper address subitem 5-ix?

Copy and paste relevant sections from the manuscript (include quotes in quotation marks "like this" to indicate direct quotes from your manuscript), or elaborate on this item by providing additional information not in the ms, or briefly explain why the item is not applicable/relevant for your study

您的回答

5-x) Clarify the level of human involvement

Clarify the level of human involvement (care providers or health professionals, also technical assistance) in the e-intervention or as co-intervention (detail number and expertise of professionals involved, if any, as well as “type of assistance offered, the timing and frequency of the support, how it is initiated, and the medium by which the assistance is delivered”. It may be necessary to distinguish between the level of human involvement required for the trial, and the level of human involvement required for a routine application outside of a RCT setting (discuss under item 21 – generalizability).

	1	2	3	4	5	
subitem not at all important	○	○	○	○	○	essential

Does your paper address subitem 5-x?

Copy and paste relevant sections from the manuscript (include quotes in quotation marks "like this" to indicate direct quotes from your manuscript), or elaborate on this item by providing additional information not in the ms, or briefly explain why the item is not applicable/relevant for your study

您的回答

5-xi) Report any prompts/reminders used

Report any prompts/reminders used: Clarify if there were prompts (letters, emails, phone calls, SMS) to use the application, what triggered them, frequency etc. It may be necessary to distinguish between the level of prompts/reminders required for the trial, and the level of prompts/reminders for a routine application outside of a RCT setting (discuss under item 21 – generalizability).

	1	2	3	4	5	
subitem not at all important	○	○	○	○	○	essential

Does your paper address subitem 5-xi? *

Copy and paste relevant sections from the manuscript (include quotes in quotation marks "like this" to indicate direct quotes from your manuscript), or elaborate on this item by providing additional information not in the ms, or briefly explain why the item is not applicable/relevant for your study

"In the feedback group, patients received real-time visual feedback of the movement time every day through the display of the ePM. According to the wards' routine care based on ERAS guidelines, patients were reminded to engage in activities combined with ePM/ep pod feedback to meet predefined daily step goals, and also informed about the gap between the active time and the target time.'
"In the control group, patients' movement time was set to not appear on the display of the ePM. Patients were not given feedback and they were only encouraged to carry out activities according to the current care standards based on ERAS quidelines."

5-xii) Describe any co-interventions (incl. training/support)

Describe any co-interventions (incl. training/support): Clearly state any interventions that are provided in addition to the targeted eHealth intervention, as ehealth intervention may not be designed as stand-alone intervention. This includes training sessions and support [1]. It may be necessary to distinguish between the level of training required for the trial, and the level of training for a routine application outside of a RCT setting (discuss under item 21 – generalizability.

	1	2	3	4	5	
subitem not at all important	○	○	○	○	○	essential

Does your paper address subitem 5-xii? *

Copy and paste relevant sections from the manuscript (include quotes in quotation marks "like this" to indicate direct quotes from your manuscript), or elaborate on this item by providing additional information not in the ms, or briefly explain why the item is not applicable/relevant for your study

There were no co-interventions in this study.

6a) Completely defined pre-specified primary and secondary outcome measures, including how and when they were assessed

Does your paper address CONSORT subitem 6a? *

Copy and paste relevant sections from the manuscript (include quotes in quotation marks "like this" to indicate direct quotes from your manuscript), or elaborate on this item by providing additional information not in the ms, or briefly explain why the item is not applicable/relevant for your study

Primary outcome
"The primary outcome was the Comprehensive Complication Index (CCI) [25] within 30 days after surgery. The CCI measured the overall complication calculated for each patient, ranging from 0 (no complication) to 100 (death), including any minor complications instead of focusing on specific ones. It was thus an objective measure with comprehensive and accurate evaluation [26-28]. When calculating CCI, all postoperative complications were evaluated and classified based on the Clavien-Dindo classification, then converted into a weighted continuous scale including quantity and severity. Finally, the CCI value was evaluated using the CCI calculator available at www.assesssurgery.com. "
Secondary outcomes
"Daily activity time was set from 0:00 to 24:00 based on the data uploaded by ePM/ep pod until the 4th day or until discharge, whichever came first. The patient's compliance was considered to be low [29] on condition that the patient wore it for less than 8 hours between 8:00 and 20:00. The degree of pain for rest and exercise in the first 3 days after operation was scored by the NRS [30]. NRS used 0-10 points to represent different degrees of pain scored by patients according to their subjective feelings, 0 for no pain and 10 for severe pain. The length of stay was set as the actual length of stay after a patient's operation. The percentage of reaching the scheduled mobilization target was calculated. The patient's activity was considered to be not up to standard if the daily activity time after their operation did not reach the target time. The mortality rate of 30 days after operation was also counted. The first exhaust and defecating times were subject to the patient's own assessment, meaning that an obvious feeling of exhaust or defecation was reported."

6a-i) Online questionnaires: describe if they were validated for online use and apply CHERRIES items to describe how the questionnaires were designed/deployed

If outcomes were obtained through online questionnaires, describe if they were validated for online use and apply CHERRIES items to describe how the questionnaires were designed/deployed [9].

	1	2	3	4	5	
subitem not at all important	○	○	○	○	○	essential

Does your paper address subitem 6a-i?

Copy and paste relevant sections from manuscript text

您的回答

6a-ii) Describe whether and how “use” (including intensity of use/dosage) was defined/measured/monitored

Describe whether and how “use” (including intensity of use/dosage) was defined/measured/monitored (logins, logfile analysis, etc.). Use/adoption metrics are important process outcomes that should be reported in any ehealth trial.

	1	2	3	4	5	
subitem not at all important	○	○	○	○	○	essential

Does your paper address subitem 6a-ii?

Copy and paste relevant sections from manuscript text

您的回答

6a-iii) Describe whether, how, and when qualitative feedback from participants was obtained

Describe whether, how, and when qualitative feedback from participants was obtained (e.g., through emails, feedback forms, interviews, focus groups).

	1	2	3	4	5	
subitem not at all important	○	○	○	○	○	essential

Does your paper address subitem 6a-iii?

Copy and paste relevant sections from manuscript text

您的回答

6b) Any changes to trial outcomes after the trial commenced, with reasons

Does your paper address CONSORT subitem 6b? *

Copy and paste relevant sections from the manuscript (include quotes in quotation marks "like this" to indicate direct quotes from your manuscript), or elaborate on this item by providing additional information not in the ms, or briefly explain why the item is not applicable/relevant for your study

There were no changes to trial outcomes after the trial commenced.

7a) How sample size was determined

NPT: When applicable, details of whether and how the clustering by care provides or centers was addressed

7a-i) Describe whether and how expected attrition was taken into account when calculating the sample size

Describe whether and how expected attrition was taken into account when calculating the sample size.

	1	2	3	4	5	
subitem not at all important	○	○	○	○	○	essential

Does your paper address subitem 7a-i?

Copy and paste relevant sections from manuscript title (include quotes in quotation marks "like this" to indicate direct quotes from your manuscript), or elaborate on this item by providing additional information not in the ms, or briefly explain why the item is not applicable/relevant for your study

"The sample size calculation was based on the primary outcome measured by the CCI 30 days after operation. CCI represents the measurement data that follows a normal distribution, as detailed in the previous trials on postoperative complications [31,32]. The average postoperative CCI of CRC was 10 (SD5). Assuming that the visual feedback of the movement time could reduce CCI by 25%, the average CCI of the feedback group was set to 7.5 (SD5) [33]. Based on the above effect size, PASS 2015 software was used to calculate that a minimum of 172 patients (86 per group) would provide 90% power to detect a 25% decrease of CCI and an α of 0.05 (two-sided test), assuming the feedback group was more favorable compared to the control group [33]. To compensate for a potential 20% drop-out rate, 216 patients were required to be included (108 per group)."

7b) When applicable, explanation of any interim analyses and stopping guidelines

Does your paper address CONSORT subitem 7b? *

Copy and paste relevant sections from the manuscript (include quotes in quotation marks "like this" to indicate direct quotes from your manuscript), or elaborate on this item by providing additional information not in the ms, or briefly explain why the item is not applicable/relevant for your study

No interim analysis was performed. The study was designed to collect complete data to evaluate the primary outcome and was designed to take into account all possible risks and ensure the safety of the participants. We monitored the progress of the study through regular data review and communication with the independent ethics committee to ensure the integrity of the study.

8a) Method used to generate the random allocation sequence

NPT: When applicable, how care providers were allocated to each trial group

Does your paper address CONSORT subitem 8a? *

Copy and paste relevant sections from the manuscript (include quotes in quotation marks "like this" to indicate direct quotes from your manuscript), or elaborate on this item by providing additional information not in the ms, or briefly explain why the item is not applicable/relevant for your study

"Patients were randomized using block random groups (sized blocks of 2 and 4) generated by specialized staff. All random assignments were made and sealed to an envelope by intervention staff using SAS software. The randomization process was performed by an independent anesthesiologist at the end of the surgical procedure, separated from the ward's nursing staff and outcome assessors to reduce potential bias."

8b) Type of randomisation; details of any restriction (such as blocking and block size)

Does your paper address CONSORT subitem 8b? *

Copy and paste relevant sections from the manuscript (include quotes in quotation marks "like this" to indicate direct quotes from your manuscript), or elaborate on this item by providing additional information not in the ms, or briefly explain why the item is not applicable/relevant for your study

"Patients were randomized using block random groups (sized blocks of 2 and 4) generated by specialized staff. All random assignments were made and sealed to an envelope by intervention staff using SAS software. The randomization process was performed by an independent anesthesiologist at the end of the surgical procedure, separated from the ward's nursing staff and outcome assessors to reduce potential bias."

9) Mechanism used to implement the random allocation sequence (such as sequentially numbered containers), describing any steps taken to conceal the sequence until interventions were assigned

Does your paper address CONSORT subitem 9? *

Copy and paste relevant sections from the manuscript (include quotes in quotation marks "like this" to indicate direct quotes from your manuscript), or elaborate on this item by providing additional information not in the ms, or briefly explain why the item is not applicable/relevant for your study

"Patients were randomized using block random groups (sized blocks of 2 and 4) generated by specialized staff. All random assignments were made and sealed to an envelope by intervention staff using SAS software. The randomization process was performed by an independent anesthesiologist at the end of the surgical procedure, separated from the ward's nursing staff and outcome assessors to reduce potential bias."

10) Who generated the random allocation sequence, who enrolled participants, and who assigned participants to interventions

Does your paper address CONSORT subitem 10? *

Copy and paste relevant sections from the manuscript (include quotes in quotation marks "like this" to indicate direct quotes from your manuscript), or elaborate on this item by providing additional information not in the ms, or briefly explain why the item is not applicable/relevant for your study

"Patients were randomized using block random groups (sized blocks of 2 and 4) generated by specialized staff. All random assignments were made and sealed to an envelope by intervention staff using SAS software. The randomization process was performed by an independent anesthesiologist at the end of the surgical procedure, separated from the ward's nursing staff and outcome assessors to reduce potential bias."

11a) If done, who was blinded after assignment to interventions (for example, participants, care providers, those assessing outcomes) and how

NPT: Whether or not administering co-interventions were blinded to group assignment

11a-i) Specify who was blinded, and who wasn’t

Specify who was blinded, and who wasn’t. Usually, in web-based trials it is not possible to blind the participants [1, 3] (this should be clearly acknowledged), but it may be possible to blind outcome assessors, those doing data analysis or those administering co-interventions (if any).

	1	2	3	4	5	
subitem not at all important	○	○	○	○	○	essential

Does your paper address subitem 11a-i? *

Copy and paste relevant sections from the manuscript (include quotes in quotation marks "like this" to indicate direct quotes from your manuscript), or elaborate on this item by providing additional information not in the ms, or briefly explain why the item is not applicable/relevant for your study

"It was impractical to blind the intervention staff in view of the intervening measure, but contrary to the outcome evaluators. To ensure blinding during outcome assessments in the surgical ward, a schedule was established to stagger the intervention and evaluation implementation. Ward staff were informed about the activity monitoring study but were not provided with any specific hypothetical details to prevent potential bias from influencing the study results. Given the intervention characteristics, blinding the patients was also deemed to be unfeasible. To minimize performance bias, patients were informed that we were evaluating two mobilization schemes on the understanding that neither scheme was expected to confer any potential advantage."

11a-ii) Discuss e.g., whether participants knew which intervention was the “intervention of interest” and which one was the “comparator”

Informed consent procedures (4a-ii) can create biases and certain expectations - discuss e.g., whether participants knew which intervention was the “intervention of interest” and which one was the “comparator”.

	1	2	3	4	5	
subitem not at all important	○	○	○	○	○	essential

Does your paper address subitem 11a-ii?

Copy and paste relevant sections from the manuscript (include quotes in quotation marks "like this" to indicate direct quotes from your manuscript), or elaborate on this item by providing additional information not in the ms, or briefly explain why the item is not applicable/relevant for your study

您的回答

11b) If relevant, description of the similarity of interventions

(this item is usually not relevant for ehealth trials as it refers to similarity of a placebo or sham intervention to a active medication/intervention)

Does your paper address CONSORT subitem 11b? *

Copy and paste relevant sections from the manuscript (include quotes in quotation marks "like this" to indicate direct quotes from your manuscript), or elaborate on this item by providing additional information not in the ms, or briefly explain why the item is not applicable/relevant for your study

"To ensure blinding during outcome assessments in the surgical ward, a schedule was established to stagger the intervention and evaluation implementation. Ward staff were informed about the activity monitoring study but were not provided with any specific hypothetical details to prevent potential bias from influencing the study results. Given the intervention characteristics, blinding the patients was also deemed to be unfeasible. To minimize performance bias, patients were informed that we were evaluating two mobilization schemes on the understanding that neither scheme was expected to confer any potential advantage."

12a) Statistical methods used to compare groups for primary and secondary outcomes

NPT: When applicable, details of whether and how the clustering by care providers or centers was addressed

Does your paper address CONSORT subitem 12a? *

Copy and paste relevant sections from the manuscript (include quotes in quotation marks "like this" to indicate direct quotes from your manuscript), or elaborate on this item by providing additional information not in the ms, or briefly explain why the item is not applicable/relevant for your study

"Continuous variables with independent access to mean and standard deviation were initially subjected to the Kolmogorov-Smirnov test to assess their normality. Otherwise, Shapiro-Wilk test was chose. Levene's test was used for the analysis of homogeneity of variance. Data with skewed distributions are reported as medians with interquartile ranges (IQR), while data adhering to a normal distribution are given as means with standard deviations (SD). Categorical variables are expressed as frequencies, percentages and ratios. For baseline comparisons, normally distributed continuous variables with comparible varience were analyzed using Student's t-test. In contrast, continuous variables with skewed distributions or uneven variance were analyzed with the Mann-Whitney U test. Categorical variables were compared using either the chi-squared or Fisher's exact tests. "
"The impact of the primary outcome CCI within 30 days after an operation was analyzed using Student's t- test or the Mann-Whitney U test."
"In analyses of secondary outcomes, the Mann-Whitney U or Student's t-tests were used to analyze the other measurement data except for repeated measurements. The chi-squared test was used to analyze the percentage of patients who achieved the predetermined mobilization goal. Binary logistic regression analysis was used to determine the determinants of patient cohorts to achieve mobilization goals. The regression model considered the following variables: age, sex, ASA grade, surgical site and the IPAQ [24]. Repeated measurement of the NRS pain score, using a generalized linear mixed model (GLMM) with multinomial distribution, was used for within-group comparisons."

12a-i) Imputation techniques to deal with attrition / missing values

Imputation techniques to deal with attrition / missing values: Not all participants will use the intervention/comparator as intended and attrition is typically high in ehealth trials. Specify how participants who did not use the application or dropped out from the trial were treated in the statistical analysis (a complete case analysis is strongly discouraged, and simple imputation techniques such as LOCF may also be problematic [4]).

	1	2	3	4	5	
subitem not at all important	○	○	○	○	○	essential

Does your paper address subitem 12a-i? *

Copy and paste relevant sections from the manuscript (include quotes in quotation marks "like this" to indicate direct quotes from your manuscript), or elaborate on this item by providing additional information not in the ms, or briefly explain why the item is not applicable/relevant for your study

"We followed the intention-to-treat (ITT) principle analyzing all random patients in the baseline analysis. The results analysis of the study were based on the modified intention-to-treat analysis (MITT) set"
"Except for MITT analysis, subjects with low device compliance (wearing ePM/ep pod for less than 8 hours from 8:00 to 20:00) were excluded for sensitivity analysis of the primary outcome."

12b) Methods for additional analyses, such as subgroup analyses and adjusted analyses

Does your paper address CONSORT subitem 12b? *

Copy and paste relevant sections from the manuscript (include quotes in quotation marks "like this" to indicate direct quotes from your manuscript), or elaborate on this item by providing additional information not in the ms, or briefly explain why the item is not applicable/relevant for your study

"We followed the intention-to-treat (ITT) principle analyzing all random patients in the baseline analysis. The results analysis of the study were based on the modified intention-to-treat analysis (MITT) set"
"In addition, subgroup analyses (pre-selected age, ASA, preoperative chemoradiotherapy, surgical site and preoperative mobility) were carried out. Simultaneously we examined the interaction between the treatment effect and these five subgroup variables. In addition, we statistically described the distribution of postoperative complications at all levels in the two groups and performed a chi-squared test between the groups. As a post hoc analysis, the Spearman/point-biserial correlation coefficient was used to determine the correlation between CCI and the postoperative movement time. In the preliminary analysis, the linear regression model showed a good fitting effect. Based on this result, we conducted a linear regression analysis to assess the relationship between the movement time of POD3 (for patients discharged on POD3, the activity time on POD2 was used to fill in the data) and CCI, adjusted for potential factors such as age, ASA, sex, preoperative chemoradiotherapy and BMI, all factors which could influence postoperative recovery. "

X26) REB/IRB Approval and Ethical Considerations [recommended as subheading under "Methods"] (not a CONSORT item)

X26-i) Comment on ethics committee approval

	1	2	3	4	5	
subitem not at all important	○	○	○	○	○	essential

Does your paper address subitem X26-i?

Copy and paste relevant sections from the manuscript (include quotes in quotation marks "like this" to indicate direct quotes from your manuscript), or elaborate on this item by providing additional information not in the ms, or briefly explain why the item is not applicable/relevant for your study

"The Ethics Committee of the First Affiliated Hospital of the Air Force Military Medical University approved this randomized controlled trial (Approval No. KY20222299-F-1), and the study protocol was registered with the Chinese Clinical Trial Registry (ChiCTR2300068107) on July 2, 2023."

x26-ii) Outline informed consent procedures

Outline informed consent procedures e.g., if consent was obtained offline or online (how? Checkbox, etc.?), and what information was provided (see 4a-ii). See [6] for some items to be included in informed consent documents.

	1	2	3	4	5	
subitem not at all important	○	○	○	○	○	essential

Does your paper address subitem X26-ii?

Copy and paste relevant sections from the manuscript (include quotes in quotation marks "like this" to indicate direct quotes from your manuscript), or elaborate on this item by providing additional information not in the ms, or briefly explain why the item is not applicable/relevant for your study

"All procedures complied with the Declaration of Helsinki and institutional regulations. Written informed consent was obtained from all participants prior to enrollment, which included detailed explanation of the study, and their right to withdraw at any time. Upon registration, each patient was assigned a unique study number, and all subsequent data analyses were performed using a de-identified dataset to ensure participant privacy and confidentiality. No financial compensation was provided for participation."

X26-iii) Safety and security procedures

Safety and security procedures, incl. privacy considerations, and any steps taken to reduce the likelihood or detection of harm (e.g., education and training, availability of a hotline)

	1	2	3	4	5	
subitem not at all important	○	○	○	○	○	essential

Does your paper address subitem X26-iii?

Copy and paste relevant sections from the manuscript (include quotes in quotation marks "like this" to indicate direct quotes from your manuscript), or elaborate on this item by providing additional information not in the ms, or briefly explain why the item is not applicable/relevant for your study

"All procedures complied with the Declaration of Helsinki and institutional regulations. Written informed consent was obtained from all participants prior to enrollment, which included detailed explanation of the study, and their right to withdraw at any time. Upon registration, each patient was assigned a unique study number, and all subsequent data analyses were performed using a de-identified dataset to ensure participant privacy and confidentiality. No financial compensation was provided for participation."
"If the following incidents occur, activity supervision should be suspended immediately: participants experience pain intensity>4(from 0 to 10), ECG abnormalities (signs of arrhythmia or acute ischemia), indoor air oxygen saturation <88%, systolic blood pressure <90mmHg, or symptoms of standing intolerance (ie, dizziness, nausea, and blurred vision), exercise weakness (ie, difficulty standing), etc. In the next scheduled mobilization, if the participant is considered to be in stable physical condition after clinical evaluation, a new mobilization attempt will be conducted. If serious adverse events such as falling out of bed or falling occur, in addition to suspending activity supervision, the competent physician should also be asked to evaluate the patient's injury and terminate the study if necessary. The researcher should immediately fill out the serious adverse event report and report it to the hospital ethics committee."

RESULTS

13a) For each group, the numbers of participants who were randomly assigned, received intended treatment, and were analysed for the primary outcome

NPT: The number of care providers or centers performing the intervention in each group and the number of patients treated by each care provider in each center

Does your paper address CONSORT subitem 13a? *

Copy and paste relevant sections from the manuscript (include quotes in quotation marks "like this" to indicate direct quotes from your manuscript), or elaborate on this item by providing additional information not in the ms, or briefly explain why the item is not applicable/relevant for your study

"A total of CRC 239 patients were recruited between February 2023 and September 2023. Of these, 206 were randomized, 108 into usual care and 108 with activity feedback. Two patients who met the exclusion criteria were inadvertently enrolled due to unreported medical histories and were subsequently excluded from the mITT analysis (Figure 1)"

13b) For each group, losses and exclusions after randomisation, together with reasons

Does your paper address CONSORT subitem 13b? (NOTE: Preferably, this is shown in a CONSORT flow diagram) *

Copy and paste relevant sections from the manuscript (include quotes in quotation marks "like this" to indicate direct quotes from your manuscript), or elaborate on this item by providing additional information not in the ms, or briefly explain why the item is not applicable/relevant for your study

"Two patients who met the exclusion criteria were inadvertently enrolled due to unreported medical histories and were subsequently excluded from the mITT analysis (Figure 1)"
"Removing 15 patients with low device compliance as a sensitivity analysis,......"

13b-i) Attrition diagram

Strongly recommended: An attrition diagram (e.g., proportion of participants still logging in or using the intervention/comparator in each group plotted over time, similar to a survival curve) or other figures or tables demonstrating usage/dose/engagement.

	1	2	3	4	5	
subitem not at all important	○	○	○	○	○	essential

Does your paper address subitem 13b-i?

Copy and paste relevant sections from the manuscript or cite the figure number if applicable (include quotes in quotation marks "like this" to indicate direct quotes from your manuscript), or elaborate on this item by providing additional information not in the ms, or briefly explain why the item is not applicable/relevant for your study

您的回答

14a) Dates defining the periods of recruitment and follow-up

Does your paper address CONSORT subitem 14a? *

Copy and paste relevant sections from the manuscript (include quotes in quotation marks "like this" to indicate direct quotes from your manuscript), or elaborate on this item by providing additional information not in the ms, or briefly explain why the item is not applicable/relevant for your study

Patients were recruited "between February 2023 and September 2023."
We followed "postoperative complications of CRC patients within 30 days after their operations."

14a-i) Indicate if critical “secular events” fell into the study period

Indicate if critical “secular events” fell into the study period, e.g., significant changes in Internet resources available or “changes in computer hardware or Internet delivery resources”

	1	2	3	4	5	
subitem not at all important	○	○	○	○	○	essential

Does your paper address subitem 14a-i?

Copy and paste relevant sections from the manuscript (include quotes in quotation marks "like this" to indicate direct quotes from your manuscript), or elaborate on this item by providing additional information not in the ms, or briefly explain why the item is not applicable/relevant for your study

您的回答

14b) Why the trial ended or was stopped (early)

Does your paper address CONSORT subitem 14b? *

Copy and paste relevant sections from the manuscript (include quotes in quotation marks "like this" to indicate direct quotes from your manuscript), or elaborate on this item by providing additional information not in the ms, or briefly explain why the item is not applicable/relevant for your study

The trial ended normally after we had enrolled enough participants to power the study.

15) A table showing baseline demographic and clinical characteristics for each group

NPT: When applicable, a description of care providers (case volume, qualification, expertise, etc.) and centers (volume) in each group

Does your paper address CONSORT subitem 15? *

Copy and paste relevant sections from the manuscript (include quotes in quotation marks "like this" to indicate direct quotes from your manuscript), or elaborate on this item by providing additional information not in the ms, or briefly explain why the item is not applicable/relevant for your study

"Patients in each randomized group had similar baseline and perioperative characteristics (Table 1)"

15-i) Report demographics associated with digital divide issues

In ehealth trials it is particularly important to report demographics associated with digital divide issues, such as age, education, gender, social-economic status, computer/Internet/ehealth literacy of the participants, if known.

	1	2	3	4	5	
subitem not at all important	○	○	○	○	○	essential

Does your paper address subitem 15-i? *

Copy and paste relevant sections from the manuscript (include quotes in quotation marks "like this" to indicate direct quotes from your manuscript), or elaborate on this item by providing additional information not in the ms, or briefly explain why the item is not applicable/relevant for your study

Not relevant to this trial. Participants were not asked to access digital materials.

16) For each group, number of participants (denominator) included in each analysis and whether the analysis was by original assigned groups

16-i) Report multiple “denominators” and provide definitions

Report multiple “denominators” and provide definitions: Report N’s (and effect sizes) “across a range of study participation [and use] thresholds” [1], e.g., N exposed, N consented, N used more than x times, N used more than y weeks, N participants “used” the intervention/comparator at specific pre-defined time points of interest (in absolute and relative numbers per group). Always clearly define “use” of the intervention.

	1	2	3	4	5	
subitem not at all important	○	○	○	○	○	essential

Does your paper address subitem 16-i? *

Copy and paste relevant sections from the manuscript (include quotes in quotation marks "like this" to indicate direct quotes from your manuscript), or elaborate on this item by providing additional information not in the ms, or briefly explain why the item is not applicable/relevant for your study

"A total of CRC 239 patients were recruited between February 2023 and September 2023. Of these, 206 were randomized, 108 into usual care and 108 with activity feedback. Two patients who met the exclusion criteria were inadvertently enrolled due to unreported medical histories and were subsequently excluded from the mITT analysis (Figure 1)"
The feedback group received the intervention of real-time visual feedback daily through the ep-pod device, while the control group did not receive feedback.

16-ii) Primary analysis should be intent-to-treat

Primary analysis should be intent-to-treat, secondary analyses could include comparing only "users", with the appropriate caveats that this is no longer a randomized sample (see 18-i).

	1	2	3	4	5	
subitem not at all important	○	○	○	○	○	essential

Does your paper address subitem 16-ii?

Copy and paste relevant sections from the manuscript (include quotes in quotation marks "like this" to indicate direct quotes from your manuscript), or elaborate on this item by providing additional information not in the ms, or briefly explain why the item is not applicable/relevant for your study

"We followed the intention-to-treat (ITT) principle analyzing all random patients in the baseline analysis. The results analysis of the study were based on the modified intention-to-treat analysis (MITT) set."

17a) For each primary and secondary outcome, results for each group, and the estimated effect size and its precision (such as 95% confidence interval)

Does your paper address CONSORT subitem 17a? *

Copy and paste relevant sections from the manuscript (include quotes in quotation marks "like this" to indicate direct quotes from your manuscript), or elaborate on this item by providing additional information not in the ms, or briefly explain why the item is not applicable/relevant for your study

Primary outcome
"As shown in Table 2, no significant differences were found in CCI within 30 postoperative days between the control group, with a median CCI of 0 (range, 0–20.90), and the activity feedback group, with a median CCI of 0 (range, 0–12.20). The estimated mean difference between the two groups was -0.59 (95% CI: -3.56 to 2.38, P = .66). Removing 15 patients with low device compliance as a sensitivity analysis, the estimated mean difference was -0.98 (95% CI: -4.07 to 2.10, P = .54). The subgroup analysis results revealed no significant interaction between the two groups on CCI within 30 days and all the subgroup variables (Figure 2)."
Secondary outcomes
"As shown in Table 4, the two groups had no significant difference in daily activity time. Similarly, there was no difference in the rate of reaching the target between groups. The usual care and activity feedback groups demonstrated no significant difference in the length of postoperative hospital stay, with a median of 4 days for both groups (P = .99), in first exhaust time (37.13 hours vs. 38.29 hours, P = .89), and in first defecating time (44.50 hours vs. 44.13 hours, P = .91) (Table 5). The results of the GLMM (Table 6) revealed that the NRS scores of rest and activity showed a declining trend over time, with a significant time effect that was independent of the patient groups (P < .001). Moreover, there were no significant interactions between the intervention and the NRS scores at rest (P = .54) or during movement (P = .89)."
"In post hoc analysis, there was a significant negative correlation between CCI within the 30th postoperative day and the activity duration on POD2 (r = -0.166, P =.02) and POD3 (r = -0.264, P=.002). A linear regression analysis showed that the movement time of POD3 was negatively correlated with CCI within the 30th postoperative day, and the standardized β was -0.025 (95% CI: -0.045 to -0.006, P = .012). Additionally, the longer the activity time of POD3 before 215 minutes, the faster the CCI decreased, however, beyond 215 minutes, the rate of the decline slowed (Figure 3). "

17a-i) Presentation of process outcomes such as metrics of use and intensity of use

In addition to primary/secondary (clinical) outcomes, the presentation of process outcomes such as metrics of use and intensity of use (dose, exposure) and their operational definitions is critical. This does not only refer to metrics of attrition (13-b) (often a binary variable), but also to more continuous exposure metrics such as “average session length”. These must be accompanied by a technical description how a metric like a “session” is defined (e.g., timeout after idle time) [1] (report under item 6a).

	1	2	3	4	5	
subitem not at all important	○	○	○	○	○	essential

Does your paper address subitem 17a-i?

Copy and paste relevant sections from the manuscript (include quotes in quotation marks "like this" to indicate direct quotes from your manuscript), or elaborate on this item by providing additional information not in the ms, or briefly explain why the item is not applicable/relevant for your study

您的回答

17b) For binary outcomes, presentation of both absolute and relative effect sizes is recommended

Does your paper address CONSORT subitem 17b? *

Copy and paste relevant sections from the manuscript (include quotes in quotation marks "like this" to indicate direct quotes from your manuscript), or elaborate on this item by providing additional information not in the ms, or briefly explain why the item is not applicable/relevant for your study

For out primary outcome, "As shown in Table 2, no significant differences were found in CCI within 30 postoperative days between the control group, with a median CCI of 0 (range, 0–20.90), and the activity feedback group, with a median CCI of 0 (range, 0–12.20). The estimated mean difference between the two groups was -0.59 (95% CI: -3.56 to 2.38, P = .66)."
"The rate of no complications accounted for 58.3% of patients in the usual care group and 60.4% in the activity feedback group, while sever complications accounted for 4.6% and 3.8% (Table 2). Both groups had the highest proportion of grade 2 complications. In addition, the incidence of urinary retention was the highest (13.9% and 10.4%) for the operative complications. The incidence rates of complications of infectious, respiratory system and cardiovascular systems were 6.5% (9.4%), 5.5% (2.8%) and 1.9% (1.8%), respectively (Table 3). In the analysis of specific complications, by Fisher's exact test, for pneumonia, the incidence rate in the routine care group was 4.6%(5 cases), and in the activity feedback group was 1.9%(2 cases), P=.45; for ileus, the incidence rate in the routine care group was 0.9%(1 case), and in the activity feedback group was 3.8%(4 cases), P=.21, indicated that the intervention had no significant effect on the incidence of these specific complications."

18) Results of any other analyses performed, including subgroup analyses and adjusted analyses, distinguishing pre-specified from exploratory

Does your paper address CONSORT subitem 18? *

Copy and paste relevant sections from the manuscript (include quotes in quotation marks "like this" to indicate direct quotes from your manuscript), or elaborate on this item by providing additional information not in the ms, or briefly explain why the item is not applicable/relevant for your study

"Removing 15 patients with low device compliance as a sensitivity analysis, the estimated mean difference was -0.98 (95% CI: -4.07 to 2.10, P = .54)."
"The subgroup analysis results revealed no significant interaction between the two groups on CCI within 30 days and all the subgroup variables (Figure 2)."
"In post hoc analysis, there was a significant negative correlation between CCI within the 30th postoperative day and the activity duration on POD2 (r = -0.166, P =.02) and POD3 (r = -0.264, P=.002). A linear regression analysis showed that the movement time of POD3 was negatively correlated with CCI within the 30th postoperative day, and the standardized β was -0.025 (95% CI: -0.045 to -0.006, P = .012)."

18-i) Subgroup analysis of comparing only users

A subgroup analysis of comparing only users is not uncommon in ehealth trials, but if done, it must be stressed that this is a self-selected sample and no longer an unbiased sample from a randomized trial (see 16-iii).

	1	2	3	4	5	
subitem not at all important	○	○	○	○	○	essential

Does your paper address subitem 18-i?

Copy and paste relevant sections from the manuscript (include quotes in quotation marks "like this" to indicate direct quotes from your manuscript), or elaborate on this item by providing additional information not in the ms, or briefly explain why the item is not applicable/relevant for your study

您的回答

19) All important harms or unintended effects in each group
(for specific guidance see CONSORT for harms)

Does your paper address CONSORT subitem 19? *

Copy and paste relevant sections from the manuscript (include quotes in quotation marks "like this" to indicate direct quotes from your manuscript), or elaborate on this item by providing additional information not in the ms, or briefly explain why the item is not applicable/relevant for your study

There were no important harms or unintended effects in each group.

19-i) Include privacy breaches, technical problems

Include privacy breaches, technical problems. This does not only include physical “harm” to participants, but also incidents such as perceived or real privacy breaches [1], technical problems, and other unexpected/unintended incidents. “Unintended effects” also includes unintended positive effects [2].

	1	2	3	4	5	
subitem not at all important	○	○	○	○	○	essential

Does your paper address subitem 19-i?

Copy and paste relevant sections from the manuscript (include quotes in quotation marks "like this" to indicate direct quotes from your manuscript), or elaborate on this item by providing additional information not in the ms, or briefly explain why the item is not applicable/relevant for your study

您的回答

19-ii) Include qualitative feedback from participants or observations from staff/researchers

Include qualitative feedback from participants or observations from staff/researchers, if available, on strengths and shortcomings of the application, especially if they point to unintended/unexpected effects or uses. This includes (if available) reasons for why people did or did not use the application as intended by the developers.

	1	2	3	4	5	
subitem not at all important	○	○	○	○	○	essential

Does your paper address subitem 19-ii?

Copy and paste relevant sections from the manuscript (include quotes in quotation marks "like this" to indicate direct quotes from your manuscript), or elaborate on this item by providing additional information not in the ms, or briefly explain why the item is not applicable/relevant for your study

您的回答

DISCUSSION

22) Interpretation consistent with results, balancing benefits and harms, and considering other relevant evidence

NPT: In addition, take into account the choice of the comparator, lack of or partial blinding, and unequal expertise of care providers or centers in each group

22-i) Restate study questions and summarize the answers suggested by the data, starting with primary outcomes and process outcomes (use)

Restate study questions and summarize the answers suggested by the data, starting with primary outcomes and process outcomes (use).

	1	2	3	4	5	
subitem not at all important	○	○	○	○	○	essential

Does your paper address subitem 22-i? *

Copy and paste relevant sections from the manuscript (include quotes in quotation marks "like this" to indicate direct quotes from your manuscript), or elaborate on this item by providing additional information not in the ms, or briefly explain why the item is not applicable/relevant for your study

"In this study, the activity incentive feedback based on a wearable device (ePM/ep pod) did not reduce the CCI of CRC patients within postoperative 30 days. The differences between groups in terms of daily activity time, length of postoperative hospital stay, intestinal function recovery and NRS pain scores were also not significant."

22-ii) Highlight unanswered new questions, suggest future research

Highlight unanswered new questions, suggest future research.

	1	2	3	4	5	
subitem not at all important	○	○	○	○	○	essential

Does your paper address subitem 22-ii?

Copy and paste relevant sections from the manuscript (include quotes in quotation marks "like this" to indicate direct quotes from your manuscript), or elaborate on this item by providing additional information not in the ms, or briefly explain why the item is not applicable/relevant for your study

"Future studies should investigate whether such incentive feedback would be effective for patients experiencing more intense surgical stimulation or possessing less ERAS education. Previous studies using step count feedback for mobility-enhancing showed conflicting"

20) Trial limitations, addressing sources of potential bias, imprecision, and, if relevant, multiplicity of analyses

20-i) Typical limitations in ehealth trials

Typical limitations in ehealth trials: Participants in ehealth trials are rarely blinded. Ehealth trials often look at a multiplicity of outcomes, increasing risk for a Type I error. Discuss biases due to non-use of the intervention/usability issues, biases through informed consent procedures, unexpected events.

	1	2	3	4	5	
subitem not at all important	○	○	○	○	○	essential

Does your paper address subitem 20-i? *

Copy and paste relevant sections from the manuscript (include quotes in quotation marks "like this" to indicate direct quotes from your manuscript), or elaborate on this item by providing additional information not in the ms, or briefly explain why the item is not applicable/relevant for your study

"This study had some limitations. First, the 216 patients were all admitted to one hospital; therefore, the conclusion still needs to be verified in more hospitals where ERAS guidelines are less practiced. The low proportion of ASA3 patients in this study may limit our comprehensive assessment of the benefits of patients with high co-morbidities from feedback from postoperative activities. Future research may consider including more ASA3 patients to better understand the response of this special population to postoperative activity feedback intervention. Second, since it was not possible to blind the patients in this study, the two groups of patients may have influenced each other. Due to practical difficulties such as high patient mobility and limited follow-up time, this study failed to collect information about the views of patients and clinicians with the wearable device and how motivated they felt by wearing this. In the future, the study can be expanded to multiple wards to achieve cluster randomization, which may reduce bias. Future research can be also combined with qualitative items to provide a more comprehensive understanding of patient and clinician experiences and perceptions of wearable devices. Third, as 96% of patients in this study underwent laparoscopic surgery, the results may not be generalized to open surgery or other types of surgery. Although our inclusion criteria were broad, we excluded patients who had difficulty with preoperative activity or were in a poor physical condition. These patients may benefit from the incentive of activity feedback. A final limitation was there may be a difference between self-reported preoperative mobility assessed by IPAQ, which was not designed for hospital patients, but for assessing physical activity in daily life, and actual quantitative mobility. "

21) Generalisability (external validity, applicability) of the trial findings

NPT: External validity of the trial findings according to the intervention, comparators, patients, and care providers or centers involved in the trial

21-i) Generalizability to other populations

Generalizability to other populations: In particular, discuss generalizability to a general Internet population, outside of a RCT setting, and general patient population, including applicability of the study results for other organizations

	1	2	3	4	5	
subitem not at all important	○	○	○	○	○	essential

Does your paper address subitem 21-i?

Copy and paste relevant sections from the manuscript (include quotes in quotation marks "like this" to indicate direct quotes from your manuscript), or elaborate on this item by providing additional information not in the ms, or briefly explain why the item is not applicable/relevant for your study

您的回答

21-ii) Discuss if there were elements in the RCT that would be different in a routine application setting

Discuss if there were elements in the RCT that would be different in a routine application setting (e.g., prompts/reminders, more human involvement, training sessions or other co-interventions) and what impact the omission of these elements could have on use, adoption, or outcomes if the intervention is applied outside of a RCT setting.

	1	2	3	4	5	
subitem not at all important	○	○	○	○	○	essential

Does your paper address subitem 21-ii?

Copy and paste relevant sections from the manuscript (include quotes in quotation marks "like this" to indicate direct quotes from your manuscript), or elaborate on this item by providing additional information not in the ms, or briefly explain why the item is not applicable/relevant for your study

您的回答

OTHER INFORMATION

23) Registration number and name of trial registry

Does your paper address CONSORT subitem 23? *

Copy and paste relevant sections from the manuscript (include quotes in quotation marks "like this" to indicate direct quotes from your manuscript), or elaborate on this item by providing additional information not in the ms, or briefly explain why the item is not applicable/relevant for your study

Chinese Clinical Trial Registry (ChiCTR2300068107)

24) Where the full trial protocol can be accessed, if available

Does your paper address CONSORT subitem 24? *

Cite a Multimedia Appendix, other reference, or copy and paste relevant sections from the manuscript (include quotes in quotation marks "like this" to indicate direct quotes from your manuscript), or elaborate on this item by providing additional information not in the ms, or briefly explain why the item is not applicable/relevant for your study

The full trial protocol has not been published but is available upon request.

25) Sources of funding and other support (such as supply of drugs), role of funders

Does your paper address CONSORT subitem 25? *

Copy and paste relevant sections from the manuscript (include quotes in quotation marks "like this" to indicate direct quotes from your manuscript), or elaborate on this item by providing additional information not in the ms, or briefly explain why the item is not applicable/relevant for your study

This research is supported by the University-funded clinical research project (2022L2213) and Hospital-funded military medical research project (JSYXZ12) to HN.

X27) Conflicts of Interest (not a CONSORT item)

X27-i) State the relation of the study team towards the system being evaluated

In addition to the usual declaration of interests (financial or otherwise), also state the relation of the study team towards the system being evaluated, i.e., state if the authors/evaluators are distinct from or identical with the developers/sponsors of the intervention.

	1	2	3	4	5	
subitem not at all important	○	○	○	○	○	essential

Does your paper address subitem X27-i?

Copy and paste relevant sections from the manuscript (include quotes in quotation marks "like this" to indicate direct quotes from your manuscript), or elaborate on this item by providing additional information not in the ms, or briefly explain why the item is not applicable/relevant for your study

No conflict of interest in this study.

About the CONSORT EHEALTH checklist

As a result of using this checklist, did you make changes in your manuscript? *

- ○ yes, major changes
- ◉ yes, minor changes
- ○ no

What were the most important changes you made as a result of using this checklist?

您的回答

How much time did you spend on going through the checklist INCLUDING making changes in your manuscript *

We spent about 3 hours on going through the checklist including making changes to the manuscript.

As a result of using this checklist, do you think your manuscript has improved? *

- (x) yes
- ( ) no
- ( ) 其他:

Would you like to become involved in the CONSORT EHEALTH group?

This would involve for example becoming involved in participating in a workshop and writing an "Explanation and Elaboration" document

- ( ) yes
- ( ) no
- ( ) 其他:

Any other comments or questions on CONSORT EHEALTH

您的回答

STOP - Save this form as PDF before you click submit

To generate a record that you filled in this form, we recommend to generate a PDF of this page (on a Mac, simply select "print" and then select "print as PDF") before you submit it.

When you submit your (revised) paper to JMIR, please upload the PDF as supplementary file.

Don't worry if some text in the textboxes is cut off, as we still have the complete information in our database. Thank you!

Final step: Click submit !

Click submit so we have your answers in our database!

提交

切勿通过 Google 表单提交密码。

此内容不是由 Google 所创建，Google 不对其作任何担保。 - 联系表单所有者 - 服务条款 - 隐私权政策

此表单看起来很可疑吗？ 报告

Google 表单
